# Protective effect of salvianolic acid B against myocardial ischemia/reperfusion injury: preclinical systematic evaluation and meta-analysis

**DOI:** 10.3389/fphar.2024.1452545

**Published:** 2024-09-11

**Authors:** Yuhan Yang, Ziyi Sun, Xiaoning Sun, Jin Zhang, Tong Tong, Xiaoxiao Zhang, Kuiwu Yao

**Affiliations:** ^1^ Guang’ anmen Hospital, China Academy of Chinese Medical Sciences, Beijing, China; ^2^ Academic Management Service, China Academy of Chinese Medical Sciences, Beijing, China

**Keywords:** animal model, salvianolic acid B(Sal B), myocardial ischaemia-reperfusion, metaanalysis, preclinical studies

## Abstract

**Background:**

Salvianolic acid B is the most abundant water-soluble component in the traditional Chinese medicine Danshen and can reduce myocardial ischemia-reperfusion (MI/R) injury through multiple targets and pathways. However, the role of SalB in protecting the myocardium from ischemia/reperfusion injury remains unclear.

**Purpose:**

To perform a preclinical systematic review and meta-analysis to assess the efficacy of Sal B in an animal model of myocardial infarction/reperfusion (MI/R) and to summarize the potential mechanisms of Sal B against MI/R.

**Methods:**

Studies published from inception to March 2024 were systematically searched in PubMed, Web of Science, Embase, China National Knowledge Infrastructure Wanfang, and VIP databases. The methodological quality was determined using the SYRCLE RoB tool. The R software was used to analyze the data. The potential mechanisms are categorized and summarized.

**Results:**

32 studies containing 732 animals were included. The results of the meta-analysis showed that Sal B reduced myocardial infarct size (*p* < 0.01), and the cardiological indices of CK-MB (*p* < 0.01), CK (*p* < 0.01), LDH (*p* < 0.01), and cTnI (*p* < 0.01) compared to the control group. In addition, Sal B increased cardiac function indices, such as LVFS (*p* < 0.01), -dp/dt max (*p* < 0.01), +dp/dt max (*p* < 0.01), and cardiac output (*p* < 0.01). The protective effects of Sal B on the myocardium after I/R may be mediated by attenuating oxidative stress and inflammation, promoting neovascularization, regulating vascular function, and attenuating cardiac myocyte apoptosis. Publication bias was observed in all the included studies. Further studies are required to elucidate the extent of the cardioprotective effects of SalB and the safety of its use.

**Conclusion:**

To the best of our knowledge, this is the first meta-analysis of Sal B in the treatment of MI/R injury, and Sal B demonstrated a positive effect on MI/R injury through the modulation of key pathological indicators and multiple signaling pathways. Further studies are needed to elucidate the extent to which SalB exerts its cardioprotective effects and the safety of its use.

**Systematic Review Registration:**

https://www.crd.york.ac.uk/PROSPERO/.

## 1 Introduction

Myocardial infarction (MI) is caused by rupture or erosion of the epicardial coronary artery, coronary artery thrombosis, or atherosclerotic plaque formation, resulting in stenosis or occlusion of the coronary artery, leading to ischemia and hypoxia in the area of the myocardium supplied by the occluded artery. Severe and persistent ischemia and hypoxia cause irreversible damage to the myocardium, leading to death (Reed et al., 2017). According to statistics, more than seven million people worldwide are diagnosed with acute myocardial infarction (AMI) each year, and approximately 2.4 million people die of acute myocardial infarction (AMI) each year in the United States alone, resulting in an enormous health and economic burden (Yeh et al., 2010). Prompt reperfusion is the only way to save ischemic myocardium in myocardial infarction. Reperfusion has been shown to limit infarct size, improve long-term myocardial function, change the healing pattern of infarcted areas, and, more importantly, reduce mortality (Hausenloy et al., 2017). Data from France showed that between 1995 and 2015, the use of reperfusion therapy increased from 82% to 49%, whereas the 1-year mortality rate of reperfusion-treated patients decreased from 11.9% to 5.9% (Puymirat et al., 2019). Much experimental and clinical evidence supports the hypothesis that reperfusion causes additional myocardial damage. Neutrophil aggregation, calcium overload or redistribution, impaired mitochondrial energy synthesis, and burst production of oxygen free radicals occur during the reperfusion of coronary blood to ischemic tissues, further inducing myocardial injury and cardiomyocyte death, known as reperfusion injury (Heusch and Gersh, 2017). This has resulted in the current clinical dilemma that the mortality and morbidity associated with heart failure due to AMI remain high despite the increased use of reperfusion and improved methods (Sánchez-Hernández et al., 2020). Therefore, there is an urgent need for an effective therapy that can target myocardial reperfusion injury and reduce the size of the myocardial infarction. Myocardial ischemia/reperfusion (MI/R) injury is a complex pathology involving several mechanisms and molecules that cause damage to the heart. However, there are currently no satisfactory therapeutic strategies for mitigating myocardial ischemia-reperfusion injury, which may be since targeting one mechanism at a time may not be sufficient to produce a robust effect in a clinical situation where many uncontrolled variables commonly coexist (Heusch, 2020). Therefore, a multitargeted agent may be a potential avenue for addressing MI/R.

Danshen, a traditional Chinese medicine, is widely used in the treatment of angina pectoris, gastric pain, arthralgia, and menstrual disorders and is the main ingredient of Compound Danshen Dripping Pills, Danshen injections, and other medicines in the clinic (Li et al., 2018). The pharmacological effects of Salvia miltiorrhiza are mainly based on water-soluble components such as salicylic acid and fat-soluble components such as tanshinone, among which salvianolic acid B(Sal B) is the water-soluble component with the highest content (Wei et al., 2023). In recent years, researchers have focused on this promising small-molecule compound, and more preclinical studies on Sal B have been conducted. These studies suggest that Sal B exerts protective effects on ischemia-reperfusion myocardium through multiple targets, mainly through its anti-inflammatory and antioxidant effects, modulation of vasodilatation and contraction, promotion of neovascularization, and regulation of cell death functions (Ho and Hong, 2011). SalB also exerts anticoagulant, hypolipidemic, and indirect cardioprotective effects. SalB is a robust and active ingredient with an multi-target promising impact; however, no SalB injections are available on the market. A comprehensive assessment and systematic review of preclinical animal studies is essential before SalB enters clinical trials. Systematic reviews and meta-analyses can not only provide important information about the possibility of translating the results of experimental animal studies into clinical practice but also validate the efficacy, safety, and optimal dosage (Ritskes-Hoitinga et al., 2014).

To the best of our knowledge, no previous review has systematically summarized the effects of SalB in MI/R models.

Therefore, we performed a quality assessment and meta-analysis of preclinical studies using SalB in animal models to form a chain of clinical evidence and provide rigorous and systematic scientific support for further clinical studies.

## 2 Methods

This review was designed and reported according to the Preferred Reporting Items for Systematic Reviews and Meta-Analyses (PRISMA) 2020 statement (Page et al., 2021). The protocol was registered in PROSPERO (registration number: CRD42024526832).

### 2.1 Search strategy

We searched the following six databases: PubMed, EMBASE, Web of Science, China’s National Knowledge Infrastructure (CNKI), WanFang, and the VIP database (VIP). The search strategy is based on the search components “Myocardial Ischemia,” “Myocardial I/R,” and “Salvianolic acid.” The full search strategy is provided in the Supplementary Table 1. The search was limited from its inception to March 2024. We also screened the reference lists of the included studies to identify additional eligible studies.

### 2.2 Study selection

To minimize bias, the inclusion criteria were as follows: ①animal model of MI/R (modeling methods include ligation of LAD, ligation-reperfusion, and injection of ISO); ②The treatment group received only Sal B at any dose and treatment modality; ③The control group received only an equivalent amount of saline or vehicle or no treatment; ④The primary outcome of animal studies was infarct size, cardiac markers (CK, CK-MB, LDH, AST, and cTnI) and echocardiogram indicators (+dp/dt max, -dp/dt max, LVEF, LVFS, and cardiac output). Secondary outcomes were serum or protein levels associated with myocardial injury and biomarkers related to Sal B mechanisms.

The exclusion criteria were as follows: ①Case report, clinical trial, review, meeting abstract; ②The treatment group that received salvianolic acid complex or received Sal B in conjunction with other treatments; ③Animals with other disease comorbidities or No MI/R model; ④*In vitro or ex vivo* studies; ⑤No predetermined outcome index; ⑥No control group.

### 2.3 Data extraction

Two reviewers independently screened the retrieved studies’ titles and/or abstracts using the search technique to identify articles that met the inclusion criteria mentioned above. They then retrieved all the texts of these possibly eligible studies and separately determined their eligibility. If they cannot agree on whether a particular study qualifies, they will be consulted with a third reviewer to resolve the dispute. To evaluate the research quality and the evidence synthesis, two impartial reviewers gathered data from the included studies using standardized pre-pilot forms.

The extracted information will include: ①first author name and year of publication; ②Specific details on the animals in each study, including species, number, sex, and body weight; ③MI/R model and the anesthetic method used to prepare the model; ④Information about Sal B treatment, including dose, method of administration, course of treatment; as well as corresponding information in the control group; ⑥The mean and standard deviation (SD) of the results. If the article presented results from many different time points or multiple doses of SalB, only data from the last time point or the highest dose group were extracted. Because some of the data were supplied only in graphical form, we attempted to contact the authors for further clarification. If we did not receive a response, we used digital ruler software to determine the value of the graph.

### 2.4 Assessment of the risk of bias

The two reviewers conduct an independent quality assessment using the SYRCLE’s RoB tool (Hooijmans et al., 2014a), and any disagreements are resolved through consultation with the third reviewer.

### 2.5 Statistical analysis

R was used for statistical analysis. Heterogeneity was determined using the Q statistical test (*p* < 0.05, considered statistically significant) and the I^2^ statistical test. Owing to the nature and diversity of animal studies, the random effects model may better reflect reality; thus, we used a random effects model for every outcome. If at least 10 independent comparisons were available, formal subgroup analyses were performed for the modeling method, dosage, method of administration, timing of administration, and species, and sensitivity analyses were performed to assess the robustness of the meta-analysis results. All results were continuous variables; therefore, standardized mean differences (SMDs) and 95% confidence intervals (95%CI) were used to express them. Publication bias was assessed using funnel plots and Egger’s regression test.

## 3 Result

### 3.1 Study selection

**FIGURE 1:**
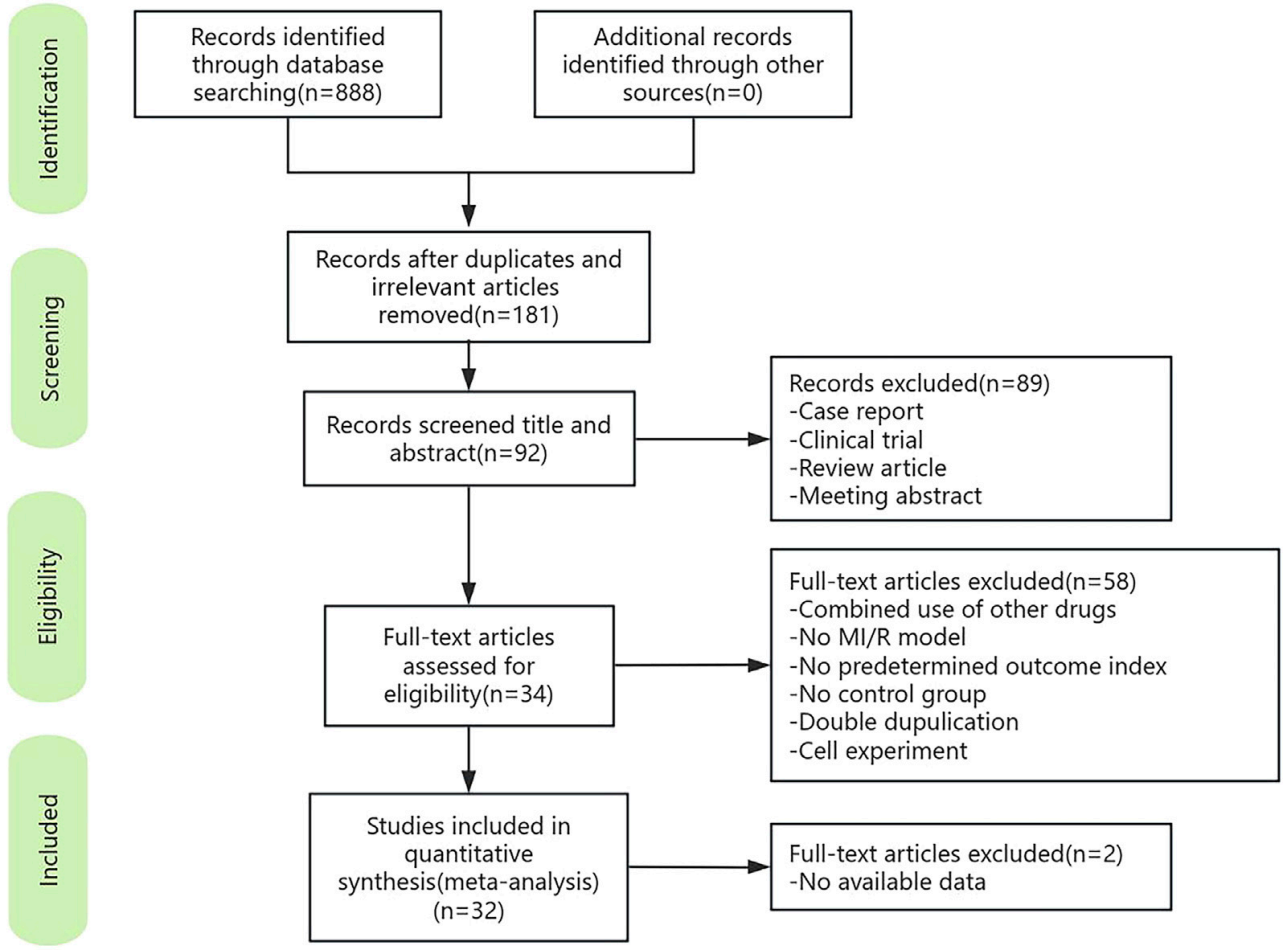
Summary of the process for identifying candidate studies.

A total of 888 articles were retrieved from the online database, 181 of which remained after duplicates were removed. 92 articles were retained after screening titles and abstracts. After full-text screening and assessment, 34 studies met the inclusion criteria. Because complete data were unavailable for two articles, 32 studies were ultimately included in this meta-analysis (Figure 1).

### 3.2 Study characteristics

Sprague-Dawley rats were used in 22 studies, Wistar rats in seven studies, ICR mice in one study, New Zealand rabbits, and 4-Way Ovoss in one study. In terms of sex, both female and male animals were used in 10 studies; one study used all female animals, one study did not explicitly report the sex of the animals, and all other studies used male animals. Fourteen of the included studies used ligation of the LAD, 13 used ligation-reperfusion, and the remaining five used ISO injection modeling. The three main routes of administration were intravenous (n = 15), intraperitoneal (n = 11), and gavage (n = 6). The dosage ranged from 3 to 120 mg/kg. In addition, the timing of Sal B administration varied, with 15 studies administering the drug prophylactically before modeling, 13 studies administering the drug after modeling or surgery, and the treatment time points of four studies were at other times. The treatment lasted 28 days Table 1 presents the characteristics of the included studies.

**TABLE 1:** Characteristics of included studies.

Study ID	Species (sex, n=experimental/control group)	Weight	Model (method)	Anesthetic	Treatment group(dosage)	Administration route	Control group	Outcomes
Qingju Li 2022 (Li et al., 2022)	Sprague–Dawley rats (male, n = 15/15)	250-300 g	Block LAD	pentobarbital sodium (30 mg/kg)	Sal B (32 mg/kg),iv	1 dose at 30 min before surgery and 1 dose at 12 h after surgery	normal saline	1)Infarct size; 2)LDH; 3)CK-MB; 4)cTnI; 5)Tunel; 6)NLRP3 mRNA and protein; 7)Caspase-1 mRNA and protein; 8)ASC mRNA and protein; 9)SIRT1 mRNA and protein; 10)AMPK α2 mRNA and protein; 11)PGC-1α mRNA and protein
Yang Hu 2019 (Hu et al., 2019)	Sprague–Dawley rats (male, n = 10/10)	220-250 g	Block LAD	3% pentobarbital sodium	Sal B (24 mg/kg),iv	1 injection immediately after surgery	normal saline	1)Infarct size; 2)LDH; 3)cTn; 4)IL-1β
Chao Lin 2016 (Lin et al., 2016)	Sprague–Dawley rats (male, n = 8/8)	200-220 g	Block LAD	chloral hydrate(0.3 g/kg)	Sal B(24 mg/kg),iv	1 dose at 24 h and 28 h after surgery	normal saline	1)Infarct size; 2)LDH; 3)CK; 4)SOD; 5)MDA; 6)Bcl-2/Bax; 7)cleaved caspase-9; 8)cleaved PARP; 9)LC3-II/LC3-I; 10)Beclin1; 11)VEGF
Lingling Xu 2011 (Xu et al., 2011)	Wistar rats (male, n = 30/30)	230-250 g	Block LAD	Not Mentioned	Sal B(10 mg/kg),iv	1 injection at 30 min and 24 h after surgery	normal saline	1)MAP; 2)-dp/dt max; 3)LVSP; 4)+dp/dt max; 5)EDP; 6)Cardiac Output; 7)Tunel; 8)PARP-1; 9)cleaved PARP-1; 10)IKKα; 11)IKKβ; 12)p-IKKa; 13)NF-κB
Xiaoying Wang 2011 (Wang et al., 2011)	Wistar rats (male,n = 15/15)	280–320 g	Block LAD	Not Mentioned	Sal B(120 mg/kg),ig	once a day for 28 consecutive days after surgery	No treatment	1)LVIDd; 2)LVIDs; 3)IVSd ; 4)IVSs; 5)LVPWd; 6)LVPWs ; 7)LVEF; 8)LVFS; 9)Infarct size
Baohong Jiang 2010 (Jiang et al., 2010)	Wistar rats (male, n = 20/20)	230-250 g	Block LAD	Not Mentioned	Sal B(10 mg/kg),iv	once a day for 14 consecutive days after surgery	normal saline	1)LVAWd; 2)LVPWd; 3)+dP/dt max; 4)-dP/dt min; 5)EDP; 6)MAP; 7)LVSP; 8)Cardiac Output; 9)Collagen volume fraction; 10)collagen I/III; 11)MMP-9; 12)MMP-2
Xiaojin Xu 2023 (Xu et al., 2023a)	Sprague–Dawley rats (male, n = 12/12)	200-250 g	Ligation for 45 min, then reperfusion for 2h	5% isoflurane	Sal B(20 mg/kg),ip	1 dose at 1 hours and 25 hour before surgery	normal saline	1)Infarct size; 2)TfR1; 3)FTH1; 4)GPX4; 5)ROS; 6)MDA; 7)LDH; 8)Tunel; 9)cleaved Caspase-3; 10)Bax; 11)Bcl-2; 12)p-MAPK; 13)p-JNK
Bo Lu 2022 (Lu et al., 2022)	Sprague–Dawley rats(female,n=10/10)	275-325 g	Ligation for 60min, then reperfusion for 27h	Not Mentioned	Sal B(60 mg/kg),iv	1 dose at 3 hours after surgery	normal saline	1)TUNEL; 2)ROS; 3)SOD; 4)MDA
Hanqing Liu 2020 (Liu et al., 2020a)	Sprague–Dawley rats (male, n = 18/18)	250-300 g	Ligation for 30min, then reperfusion for 24h	10% chloral hydrate (350 mg/kg)	Sal B(60 mg/kg),ip	once a day for 4 consecutive days before surgery	No treatment	1)Infarct size; 2)Cardiac Output; 3)LVEF; 4)LVFS; 5)Stroke Volume; 6)Heart Rate; 7)LDH; 8)CK-MB; 9)TNF-α; 10)IL-18; 11)IL-1β; 12)HMGB1; 13)TUNEL;14)Bax; 15)Bcl-2; 16)p-AKT; 17)T-AKT
Zengyong Qiao 2016 (Qiao and Xu, 2016)	Sprague–Dawley rats(male, n = 10/10)	200-250 g	Ligation for 60min, then reperfusion for 3h	ketamine hydrochloride (90 mg/kg) and xylazine hydrochloride (10 mg/kg)	Sal B(30 mg/kg),ig	once a day for 20 consecutive days before surgery	normal saline	1)LVEDV; 2)LVEDP; 3)+dp/dt max; 4)-dp/dt min; 5)MDA; 6)SOD; 7)CAT; 8)GSH-Px; 9)TNF-α; 10)IL-1β; 11)Infarct size; 12)Caspase-3; 13)Bax; 14)Bcl-2
Ling Xue 2014 (Xue et al., 2014)	Sprague–Dawley rats(male, n = 10/10)	170-190 g	Ligation for 30min, then reperfusion for 1h	20% urethane(1g/kg)	Sal B(60 mg/kg),iv	No detailed	No treatment	1)Infarct size; 2)cTnI; 3)CK-MB; 4)NO; 5)ET; 6)SOD; 7)MDA; 8)TUNEL
Zengyong Qiao 2011 (Qiao et al., 2011)	Wistar rats of (male and female, n = 10/10)	220-280 g	Ligation for 15min, then reperfusion for 2h	pentobarbitone sodium (60 mg/kg)	Sal B(55 mg/kg),ig	once a day for 12 consecutive days before surgery	distilled water	1)Infarct size; 2)AST; 3)LDH; 4)CK-MB; 5)ROS; 6)NOS; 7)MDA; 8)GSH; 9)SOD; 10)CAT; 11)GSH-Px activities
Qi Chen 2023 (Chen et al., 2023)	ICR mice (male, n = 5/5)	20-25 g	ISO	_	Sal B(10 mg/kg),ip	once a day for 7 consecutive days before modelling	No treatment	1)LDH; 2)AST; 3)CK; 4)Ca2+; 5)Bax; 6)Bcl-2; 7)NO; 8)NOS; 9)eNOS; 10)PDGF; 11)VEGF; 12)Atg5; 13)CD31; 14)LC3Ⅰ; 15)LC3Ⅱ; 16)Beclin1; 17)P62
Yang Hu 2020 (Hu et al., 2020)	Sprague–Dawley rats (male, n = 10/10)	180-220 g	ISO	_	Sal B(15 mg/kg),ip	once a day for 7 consecutive days before modelling	normal saline	1)CK-MB; 2)IL-1β; 3)NLRP3 mRNA; 4)ASC mRNA; 5)Caspase-1 mRNA; 6)IL-1β mRNA
Jun Liu 2018 (Liu et al., 2018)	Sprague–Dawley rats (male, n = 8/8)	240-260 g	ISO	_	Sal B(20 mg/kg),ip	once a day for 28 consecutive days before modelling	No treatment	1)Heart-to-body weight; 2)MDA; 3)SOD; 4)CAT; 5)GPx; 6)cTnT; 7)CK-MB; 8)LDH; 9)TNF-α; 10)NF-κB; 11)IL-1β; 12)IL-6; 13)MAP
Dan Zhou 2018 (Zhou et al., 2018)	Sprague–Dawley rats (male, n = 10/10)	250-270 g	Block LAD	Not Mentioned	Sal B(60 mg/kg), iv	once a day for 7 consecutive days after surgery	normal saline	1)Infarct size; 2)CK-MB; 3)cTnI; 4)LDH; 5)VEGF; 6)Nrf2; 7)HO-1
Dan Zhou 2013 (Zhou et al., 2013)	Sprague–Dawley rats (male, n = 10/10)	250-270 g	Ligation for 30min, then reperfusion for 3h	3% pentobarbital sodium	Sal B(60 mg/kg),iv	1 injection at the start of reperfusion	normal saline	1)Infarct size; 2)CK-MB; 3)LDH; 4)p-Akt/Akt; 5)p-eNOS/eNOS。
Guifeng Zhao 2004 (Zhao et al., 2004)	Wistar rats (male and female, n = 12/12)	200-300 g	Ligation for 30min, then reperfusion for 2h	3% pentobarbital sodium(30 mg/kg,ip)	Sal B(100 mg/kg),ig	once a day for 4 consecutive days before surgery	normal saline	1)SOD; 2)CK; 3)LDH
Qiang Zhang 2013 (Zhang et al., 2013)	Sprague–Dawley rats (male and female, n = 10/10)	200-300 g	Block LAD	2% pentobarbital sodium(40 mg/kg,ip)	Sal B(120 mg/kg),ip	once a day for 7 consecutive days before surgery	distilled water	1)CK; 2)AST; 3)LDH; 4)IL-1β; 5)IL-6; 6)TNF-α; 7)TUNEL
Fuguo Yang 2008 (Yang et al., 2008)	New Zealand rabbits (male, n = 8/8)	3.25±0.56 kg	Ligation for 30min, then reperfusion for 4h	20%urethane(5 ml/kg,iv)	Sal B(3 mg/kg),iv	1 injection after surgery	normal saline	1)NO; 2)ET
Jiangping Xu 2003 (Xu et al., 2003)	Sprague–Dawley rats (male, n = 10/10)	250-300 g	Ligation for 10min, then reperfusion for 0.5h	pentobarbital sodium(45 mg/kg,ip)	Sal B(15 mg/kg),iv	1 injection after 10 min of ischaemia	normal saline	1)Infarct size; 2)CK
Yang Xia 2018 (Xia et al., 2018)	Sprague–Dawley rats (male and female, n = 20/20)	180-220 g	Ligation for 30min, then reperfusion for 2h	Not Mentioned	Sal B(20 mg/kg), ip	once a day for 7 consecutive days before surgery	normal saline	1)Infarct size; 2)CK; 3)LDH; 4)TNF-α; 5)IL-1β; 6)ICAM-1
Baohe Wang 2004 (Wang et al., 2004)	Wistar rats (male and female, n = 12/12)	200-300 g	Ligation for 30min, then reperfusion for 2h	3% pentobarbital sodium(30 mg/kg,ip)	Sal B (100 mg/kg), ig	once a day for 4 consecutive days before surgery	normal saline	1)SOD; 2)AT-1; 3)ET; 4)TNF-α; 5)PGI2; 6)TXB2
Xinyu Wang 2016 (Wang et al., 2016)	Sprague–Dawley rats (male, n = 10/10)	180-220 g	ISO	—	Sal B(15 mg/kg),ip	once a day for 7 consecutive days before modelling	normal saline	1)CK; 2)GOT; 3)LDH; 4)MDA; 5)SOD; 6)IL-1β; 7)NLRP3; 8)Caspase-1 P20
Yanping Song 2007 (Song et al., 2007)	Sprague–Dawley rats (male and female, n = 8/8)	160-190 g	Block LAD	urethane(ip)	Sal B(10 mg/kg),iv	1 dose at 15 min after surgery	normal saline	1)Infarct size; 2)CK; 3)LDH
Chang Liu 2022 (Liu et al., 2022)	Sprague–Dawley rats (Not mentioned, n = 12/12)	Not mentioned	Block LAD	pentobarbital sodium(40 mg/kg,ip)	Sal B (40 mg/kg),ip	Started 2 days after surgery and administered once a day for 28 consecutive days	normal saline	1)CK-MB; 2)cTnI; 3)LDH; 4)ROS; 5)MDA; 6)GSH
Zhirong Lin 2011 (Lin et al., 2011)	Sprague–Dawley rats (male, n = 10/10)	240-260 g	Ligation for 30min, then reperfusion for 3h	20% urethane(0.6 ml/100g,ip)	Sal B(64 mg/kg), iv	1 injection before surgery	vehicle(5% glucose solution)	Infarct size
Lin Li 2004 (Li et al., 2004)	4-Way Ovoss(male and female, n = 5/5)	9-14 kg	Block LAD	3% pentobarbital sodium(30 mg/kg,ip)	Sal B (10 mg/kg),iv	1 injection after surgery	normal saline	1)Infarct size; 2)LDH
Xuguang Hu 2010 (Hu et al., 2010)	Sprague–Dawley rats (male and female, n = 10/10)	180-220 g	ISO	—	Sal B(10 mg/kg),ip	once a day for 5 consecutive days before modelling	normal saline	1)LDH; 2)CK; 3)SOD; 4)MDA
Yingchang Fan 2004 (Fan et al., 2004)	Wistar rats (male and female, n = 10/10)	200-300 g	Block LAD	3% pentobarbital sodium(30 mg/kg,ip)	Sal B(100 mg/kg), ig	once a day for 6 consecutive days before surgery	normal saline	Infarct size
Hengxia Chen 2012 (Chen et al., 2012)	Sprague–Dawley rats (male and female, n = 10/10)	180-220 g	Block LAD	3% pentobarbital sodium(30 mg/kg,ip)	Sal B(6.4 mg/kg), iv	once a day for 14 consecutive days after surgery	normal saline	1)Infarct size; 2)NO; 3)NOS; 4)VEGF
Yuehong Shen 2022 (Shen et al., 2022)	Sprague–Dawley rats (male, n = 8/8)	220-250 g	Block LAD	isoflurane inhalation	Sal B(50 mg/kg), ip	once a day for 14 consecutive days after surgery	No treatment	1)Infarct size; 2)LDH; 3)CK; 4)CK-MB; 5)MDA; 6)GSH; 7)LVEF; 8)LVFS; 9)Nrf2

Note: +dp/dt max, maximal left ventricular pressure rising rate; -dp/dt max, maximal left ventricular pressure decreasing rate; Bcl-2, B-cell lymphoma-2; CAT, catalase; CK, creatine kinase; CK-MB, creatine kinase isoenzymes MB; cTnI, cardiac troponin I; cTnT, cardiac troponin T; eNOS, endothelial nitric oxide synthase; GPx, myocardial glutathione peroxidase; GSH, glutathione; IL, interleukin; ISO, isoproterenol; IVSd, end-diastolic interventricular septum; IVSs, end-systolic interventricular septum; LAD, left anterior descending branch; LC3, microtuble-associated protein light chain 3; LDH, lactic dehydrogenase; LVAWd, left ventricular anterior wall thickness at diastole; LVEDP, left ventricular end dilated pressure; LVEDV, left ventricular end-diastolic volume; LVEF, left ventricular ejection fraction; LVFS, left ventricular fraction shortening; LVIDd, left ventricular internal dimension in end diastole; LVIDs, left ventricular internal dimension in end systole; LVPWd, Left ventricular posterior wall end diastole; LVPWs, Left ventricular posterior wall end systole; LVSP, left ventricular systolic pressure; MAP, mean arterial pressure; MDA, malondialdehyde; MMP, matrix metalloproteinase; NF-κB, nuclear factor kappa B; NOS, nitric oxide synthase; Nrf2, NF-E2-related factor-2; p-AKT, phosphorylated protein kinase B; PDGF, platelet derived growth factor; ROS, reactive oxygen species; SIRT1, sirtuin1; SOD, superoxide dismutase; TNF, tumor necrosis factor; TUNEL, the terminal deoxynucleotidyl transferase-mediated dUTP nick end labeling; VEGF, vascular endothelial-derived growth factor.

### 3.3 Risk of bias

Overall, 64.69% of the criteria were marked as “unclear” because basic information on the methodology was missing. For six criteria, all studies scored “unclear,” namely, Sequence generation (selection bias); Baseline characteristics (selection bias); Allocation concealment (selection bias); Blinding investigators (performance bias); Random outcome assessment (measurement bias); Blinding outcome assessor (measurement bias). Among these studies, 53.12% reported that animals were randomly grouped and housed in rooms with the same controlled temperature and moderation. One study had a high risk of bias in selective reporting because the results were not fully reported. Eight studies did not report complete data and were therefore considered high-risk. Given the high number of “unclear” scores, none of the studies were considered to be at low risk of bias. The percentage of inter-evaluator agreement for the risk of bias assessment was 79.6%. The results of the quality assessment of the included studies are shown in Table 2.

**TABLE 2:** Risk of bias for inclusion of studies.

Study ID	A	B	C	D	E	F	G	H	I	J
Qingju Li2022	?	?	?	?	?	?	?	+	-	-
Yang Hu2019	?	?	?	-	?	?	?	+	-	-
Chao Lin2016	?	?	?	-	?	?	?	-	-	-
Lingling Xu2011	?	?	?	?	?	?	?	-	-	-
Xiaoying Wang2011	?	?	?	?	?	?	?	-	-	-
Baohong Jiang2010	?	?	?	?	?	?	?	-	-	-
Xiaojin Xu2023	?	?	?	-	?	?	?	+	-	-
Bo Lu2022	?	?	?	-	?	?	?	-	-	-
Hanqing Liu2020	?	?	?	-	?	?	?	-	-	-
Zengyong Qiao2016	?	?	?	-	?	?	?	-	+	-
Ling Xue2014	?	?	?	?	?	?	?	-	-	-
Zengyong Qiao2011	?	?	?	-	?	?	?	-	-	-
Qi Chen2023	?	?	?	-	?	?	?	+	-	-
Yang Hu2020	?	?	?	-	?	?	?	-	-	-
Jun Liu2018	?	?	?	-	?	?	?	-	-	-
Dan Zhou2018	?	?	?	?	?	?	?	-	-	-
Dan Zhou2013	?	?	?	-	?	?	?	-	-	-
Guifeng Zhao2004	?	?	?	?	?	?	?	-	-	-
Qiang Zhang2013	?	?	?	-	?	?	?	-	-	-
Fuguo Yang2008	?	?	?	?	?	?	?	-	-	-
Jiangping Xu2003	?	?	?	?	?	?	?	-	-	-
Yang Xia2018	?	?	?	?	?	?	?	-	-	-
Baohe Wang2004	?	?	?	?	?	?	?	+	-	-
Xinyu Wang2016	?	?	?	-	?	?	?	+	-	-
Yanping Song2007	?	?	?	?	?	?	?	+	-	-
Chang Liu2022	?	?	?	-	?	?	?	-	-	-
Zhirong Lin2011	?	?	?	?	?	?	?	+	-	-
Lin Li2004	?	?	?	?	?	?	?	-	-	-
Xuguang Hu2010	?	?	?	-	?	?	?	-	-	-
Yingchang Fan2004	?	?	?	-	?	?	?	-	-	-
Hengxia Chen2012	?	?	?	?	?	?	?	-	-	-
Yuehong Shen2022	?	?	?	-	?	?	?	-	-	-

A, Sequence generation (selection bias); B, Baseline characteristics (selection bias); C, Allocation concealment (selection bias); D, Random housing (performance bias); E, Blinding investigators (performance bias); F,Random outcome assessment (measurement bias); G, Blinding outcome assessor (measurement bias); H, Incomplete outcome data (attrition bias); I, Selective outcome reporting (reporting bias); J, other sources of bias.

### 3.4 Outcome measures

#### 3.4.1 Infarct size

**FIGURE 2:**
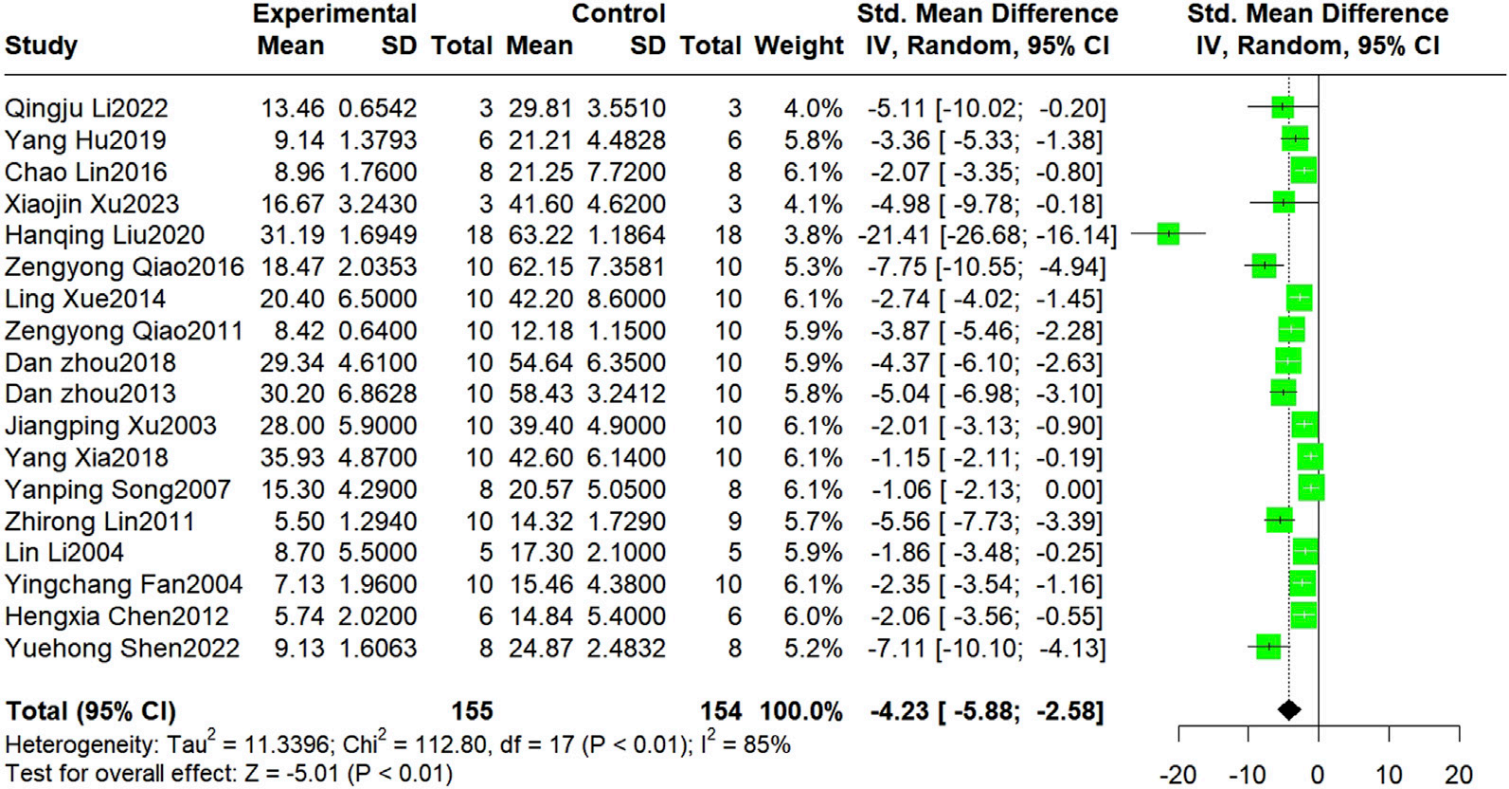
The forest plot: effect of Sal B for reducing the infarction size compared with the control group.

Nineteen studies reported the infarct size, but data were unavailable for one of these studies. Finally, 18 studies involving 309 animals were included in the meta-analysis. The results showed that Sal B was effective in reducing myocardial infarct size compared to the control group (SMD = −4.23, 95%CI(-5.88, −2.58), *p* < 0.01, I^2^ = 85%) (Figure 2). However, there was significant heterogeneity across the studies; therefore, sensitivity and subgroup analyses were conducted to explore the potential sources of heterogeneity. Sensitivity analyses were conducted by omitting individual studies that showed reliable results for infarct size (Supplementary Figure 1). Subsequently, separate subgroup analyses were performed based on modeling, administration method, time, dosage, and species (Table 3). The results showed that only the dosage subgroups were statistically significant. In addition, a substantial reduction in heterogeneity was found when blocking the LAD and Intravenous injection. Lower heterogeneity was likewise found in the dose less than 20 mg/kg dose subgroup, other administration time subgroup, and Wistar rat subgroup, but this was not statistically significant.

**TABLE 3:** Subgroup analysis of Infarct size.

Subgroup	No.of studies	SMD	95%CI	*p*-Value between subgroups	Heterogeneity within subgroups	
					I^2^ (%)	*p*-Value
Model				0.12		
Block LAD	9	−2.79	−3.80, −1.78		66%	<0.01
Ligation and reperfusion	9	−5.72	−9.25, −2.18		91%	<0.01
Dosage				<0.01		
≤20 mg/kg	6	−1.55	−2.08, −1.03		0%	0.46
21–50 mg/kg	5	−4.84	−7.19, −2.49		80%	<0.01
>50 mg/kg	7	−6.12	−10.51, −1.73		89%	<0.01
Administration method				0.29		
Intravenous injection	11	−2.9	−3.76, −2.03		66%	<0.01
gavage	3	−4.43	−7.40, −1.46		84%	<0.01
intraperitoneal injection	4	−8.45	−16.96, 0.05		96%	<0.01
Administration time				0.34		
before modeling	7	−6.42	−11.10, −1.74		93%	<0.01
after modeling	7	−2.83	−4.09, −1.57		73%	<0.01
other times	4	−3.26	−4.79, −1.72		62%	0.05
Species				0.11		
SD rats	15	−4.66	−6.74, −2.58		87%	<0.01
Wistar rats	2	−3.01	−4.49, −1.54		55%	0.13
4-way ovoss	1	−1.86	−3.48, −0.25		-	-

#### 3.4.2 Cardiac markers

**FIGURE 3:**
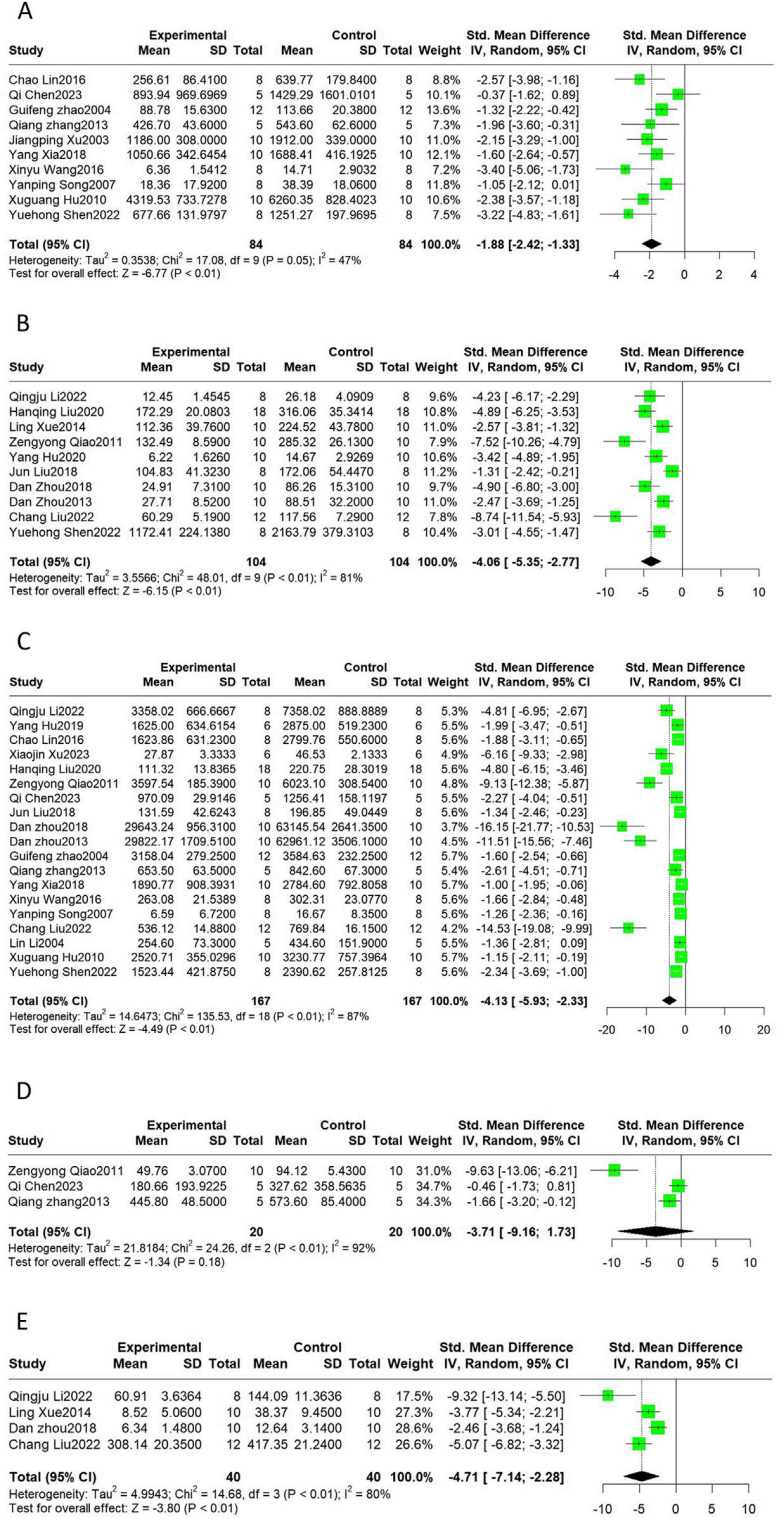
The forest plot of cardiac markers: effects of Sal B for decreasing CK **(A)**, CK-MB **(B)**, LDH **(C)**, cTnI **(E)** compared with the control group. No significant effect of Sal B for AST **(D)** compared with the control group.

CK, CK-MB, LDH, AST, and cTnI are common markers of myocardial injury and are often measured in combination to monitor the extent of myocardial injury in clinical practice. This meta-analysis indicated that CK (SMD = −1.88, 95%CI(-2.42, −1.33), *p* < 0.01, I^2^ = 47%), CK-MB (SMD = −4.06, 95%CI(-5.35, −2.77), *p* < 0.01, I^2^ = 81%), LDH (SMD = −4.13, 95%CI(-5.93, −2.33), *p* < 0.01, I^2^ = 87%), and cTnI (SMD = −4.71, 95%CI(-7.14, −2.28), *p* < 0.01, I^2^ = 80%) were reduced after the Sal B intervention. The SMD for the treatment effect of Sal B on AST was not statistically significant (SMD = −3.71, 95%CI(-9.16, 1.73), *p* = 0.18, I^2^ = 92%) (Figure 3).

Heterogeneity was high for the latter four indicators, but only LDH had more than ten publications; thus, we performed sensitivity and subgroup analyses only for LDH. By progressively eliminating each trial from the meta-analysis, no significant differences were observed between the pre-and post-sensitivity pooled effects of LDH (Supplementary Figure 2). The results of the subgroup analyses showed that the differences between subgroups were not statistically significant (Table 4). Heterogeneity was reduced in the ISO group and the subgroup treated with doses less than 20 mg/kg; however, the difference was not statistically significant.

**TABLE 4:** Subgroup analysis of LDH.

Subgroup	No.of studies	SMD	95%CI	*p*-Value between subgroups	Heterogeneity within subgroups	
					I^2^ (%)	*p*-Value
Model				0.01		
Block LAD	9	−4.75	−8.13, −1.37		87%	<0.01
Ligation and reperfusion	6	−5.37	−8.59, −2.15		92%	<0.01
ISO	4	−1.45	−2.04, −0.87		0%	0.71
Dosage				0.01		
≤20 mg/kg	8	−1.41	−1.84, −0.98		35%	0.15
21–50 mg/kg	5	−4.76	−9.01, −0.51		88%	<0.01
>50 mg/kg	6	−7.19	−11.48, −2.90		92%	<0.01
Administration method				0.69		
Intravenous injection	7	−5.1	−9.07, −1.13		89%	<0.01
gavage	2	−5.2	−12.56, 2.17		95%	<0.01
intraperitoneal injection	10	−3.38	−5.35, −1.36		86%	<0.01
Administration time				0.22		
before modeling	10	−2.83	−4.22, −1.45		83%	<0.01
after modeling	7	−5.21	−9.78, −0.65		89%	<0.01
other times	2	−7.93	−14.47, −1.39		88%	<0.01
Species				0.06		
SD rats	16	−4.68	−6.84, −2.52		89%	<0.01
ICR mice	1	−2.27	−4.04, −0.51		-	-
Wistar rats	1	−1.60	−2.54, −0.66		-	-
4-way ovoss	1	−1.36	−2.81, 0.09		-	-

#### 3.4.3 Cardiac function

**FIGURE 4:**
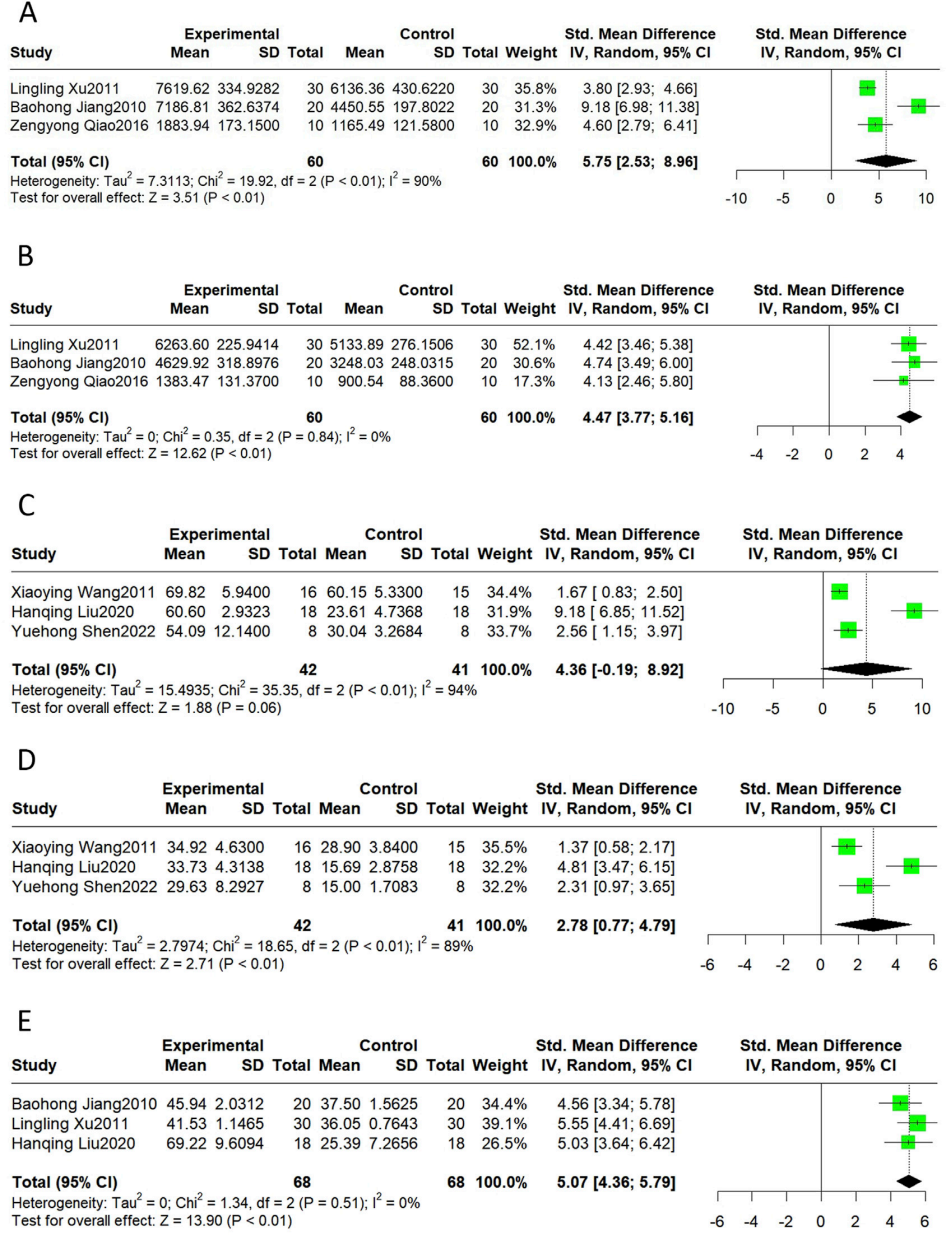
The forest plot of cardiac function: effects of Sal B for increasing + dp/dt max **(A)**, -dp/dt max **(B)**, LVFS **(D)**, Cardiac Output **(E)** compared with the control group. No significant effect of Sal B for LVEF **(C)** compared with the control group.

Echocardiographic indicators, such as + dp/dt max, -dp/dt max, LVEF, LVFS, and cardiac output, can describe changes in cardiac function and structure in MI/R models. The results of this meta-analysis suggest that Sal B had a significant effect in increasing + dp/dt max (SMD = 5.75, 95%CI(2.53, 8.96), *p* < 0.01, I^2^ = 90%), -dp/dt max (SMD = 4.47, 95%CI(3.77, 5.16), *p* < 0.01, I^2^ = 0%), LVFS(SMD = 2.78, 95%CI(0.77, 4.79), *p* < 0.01, I^2^ = 89%), and cardiac output (SMD = 5.07, 95%CI(4.36, 5.79), *p* < 0.01, I^2^ = 0%) compared to the control group (Figure 4). However, Sal B had no apparent effect on LVEF (SMD = 4.36, 95%CI(-0.19, 8.92), *p* = 0.06, I^2^ = 94%).

#### 3.4.4 Oxidative stress

**FIGURE 5:**
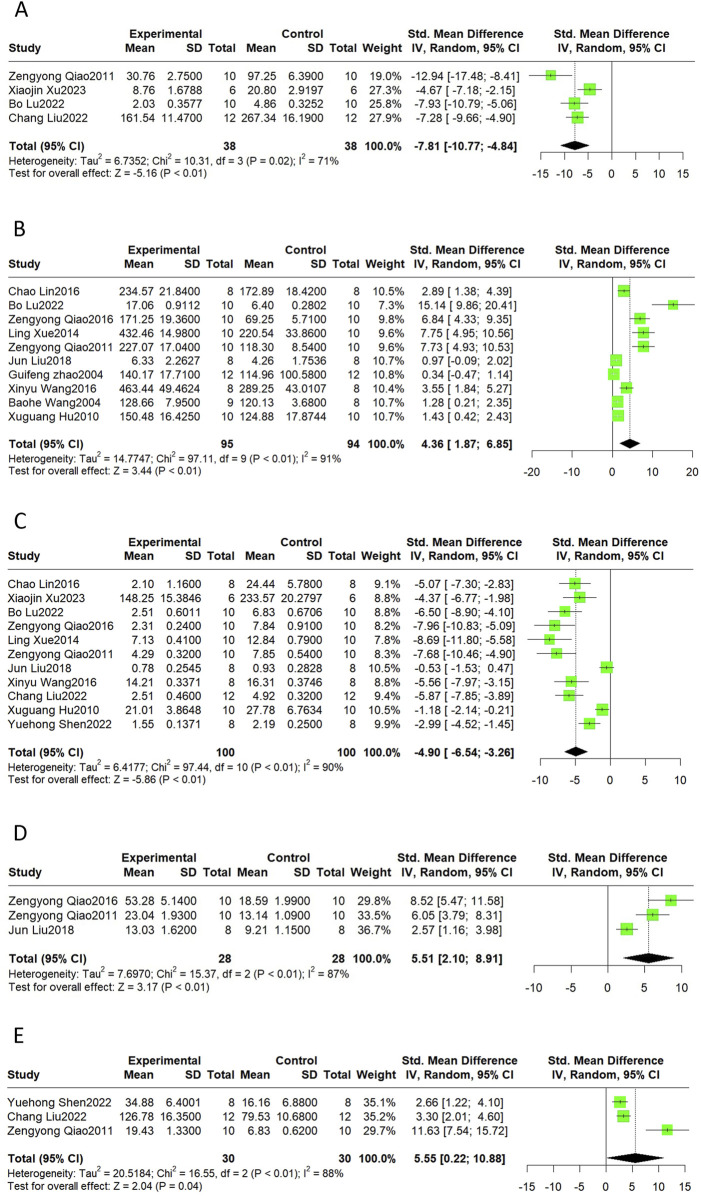
The forest plot of oxidative stress indicators: effects of Sal B for decreasing ROS **(A)**, MDA **(C)**, and increasing SOD **(B)**, CAT **(D)**, and GSH **(E)** compared with the control group.

Oxidative stress can be measured using reactive oxygen species (ROS), oxidative products, and antioxidant enzymes. MDA levels indicate the degree of lipid peroxidation. SOD and CAT are antioxidant enzymes that scavenge free radicals and prevent oxidative damage. Summarizing the available reports on the cardioprotective effects of Sal B, it was reported that Sal B intervention reduced ROS (SMD = −7.81, 95%CI(-10.77, −4.84), *p* < 0.01, I^2^ = 71%) and MDA (SMD = −4.90 95%CI(-6.54, −3.26), *p* < 0.01, I^2^ = 90%) levels and increased SOD (SMD = 4.36, 95%CI(1.87, 6.85), *p* < 0.01, I^2^ = 91%), CAT (SMD = 5.51, 95%CI(2.10, 8.91), *p* < 0.01, I^2^ = 87%) and GSH (SMD = 5.55, 95%CI (0.22, 10.88), *p =* 0.04, I^2^ = 88%) levels as compared to the control group (Figure 5). These results suggest that Sal B is a cardioprotective agent by restoring the oxidative-antioxidant balance.

#### 3.4.5 Inflammatory biomarkers

**FIGURE 6:**
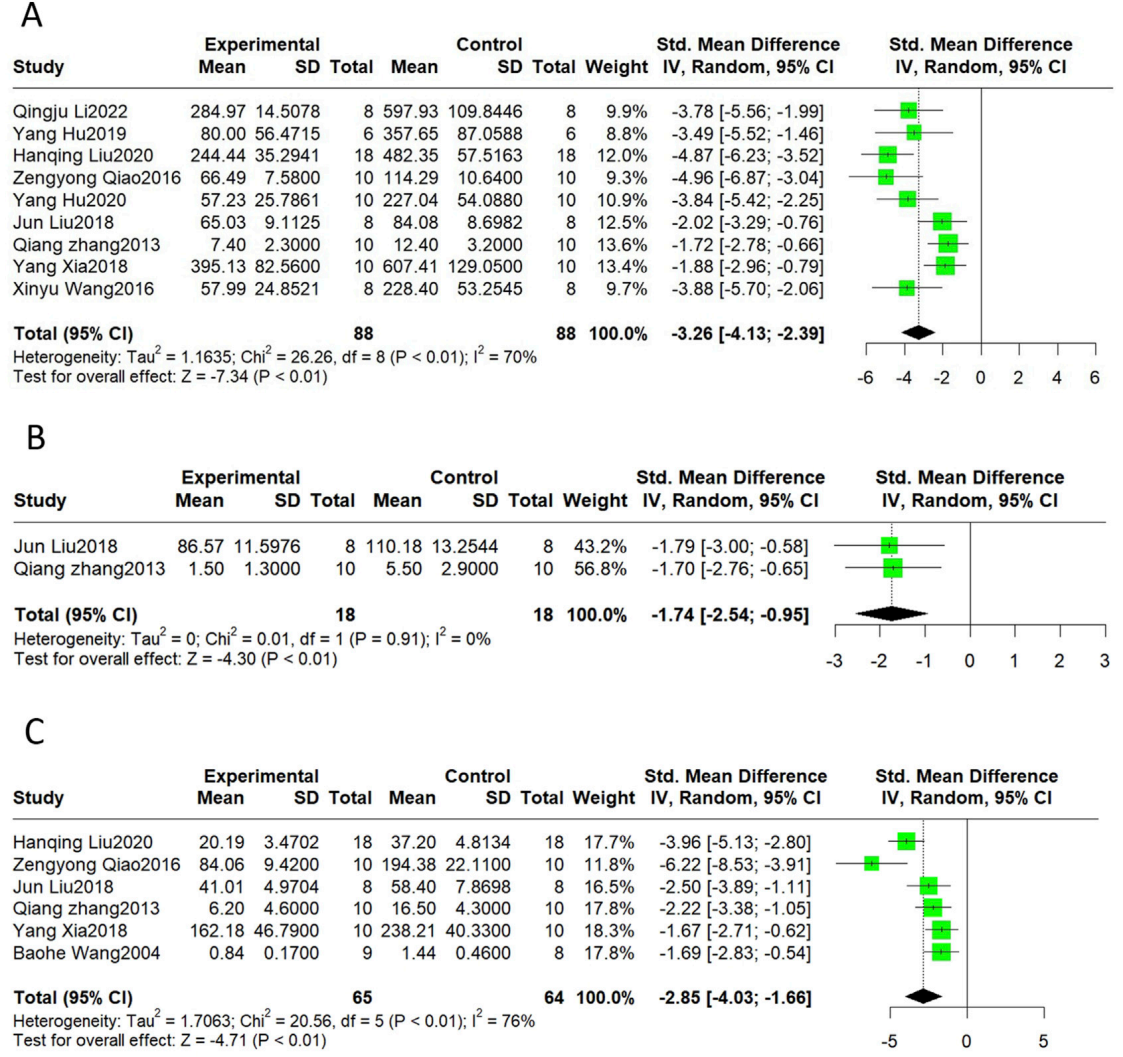
The forest plot of inflammation indicators: effects of Sal B for decreasing IL-1β **(A)**, IL-6 **(B)**, TNF-α **(C)** compared with the control group.

Overexpression of inflammatory markers is an indication of MI/R. It is associated with myocardial injury in the early stages of ischemia/reperfusion and myocardial repair after injury. The results of this meta-analysis revealed that Sal B alleviated the expression of inflammatory factors including IL-1β(SMD = −3.26, 95%CI(-4.13, −2.39), *p* < 0.01, I^2^ = 70%), IL-6(SMD = −1.74, 95%CI(-2.54, −0.95), *p* < 0.01, I^2^ = 0%) and TNF-α(SMD = −2.85 95%CI(-4.03, −1.66), *p* < 0.01, I^2^ = 76%) (Figure 6).

#### 3.4.6 Neovascularization and vasoregulation

**FIGURE 7:**
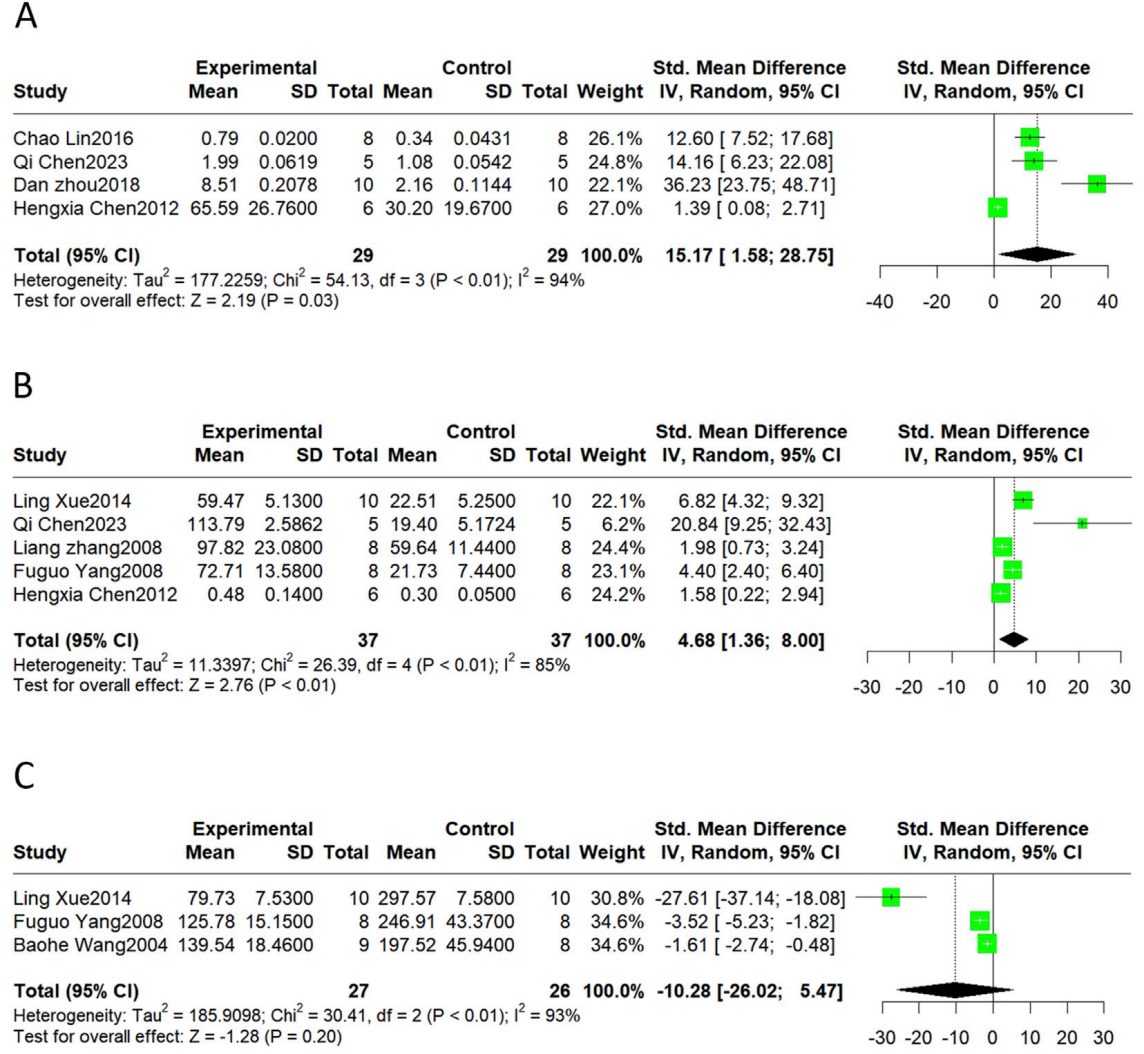
The forest plot of neovascularization and vasoregulation: effects of Sal B for increasing VEGF **(A)**, NO **(B)** compared with the control group. No significant effect of Sal B for ET **(C)** compared with the control group.

Neovascularization is an important compensatory mechanism after MI, and the vascular endothelial growth factor (VEGF) is a major biological mediator of angiogenesis *in vivo*. Promoting normal vasodilatory and contractile functions is likewise one of the important methods for alleviating MI/R injury. NO and ET are the vasodilatory and vasoconstrictive factors, respectively. Therefore, the VEGF, NO, and ET levels were considered in this meta-analysis. The results showed that SalB treatment significantly increased VEGF (SMD = 15.17, 95%CI(1.58, 28.75), *p* = 0.03, I^2^ = 94%) and NO levels (SMD = 4.68, 95%CI(1.36, 8.00), *p* < 0.01, I^2^ = 85%). Meta-analyses showed no significant effect on ET (SMD = −10.28, 95%CI(-26.02, 5.47), *p* = 0.20, I^2^ = 93%) (Figure 7).

#### 3.4.7 Apoptosis

**FIGURE 8:**
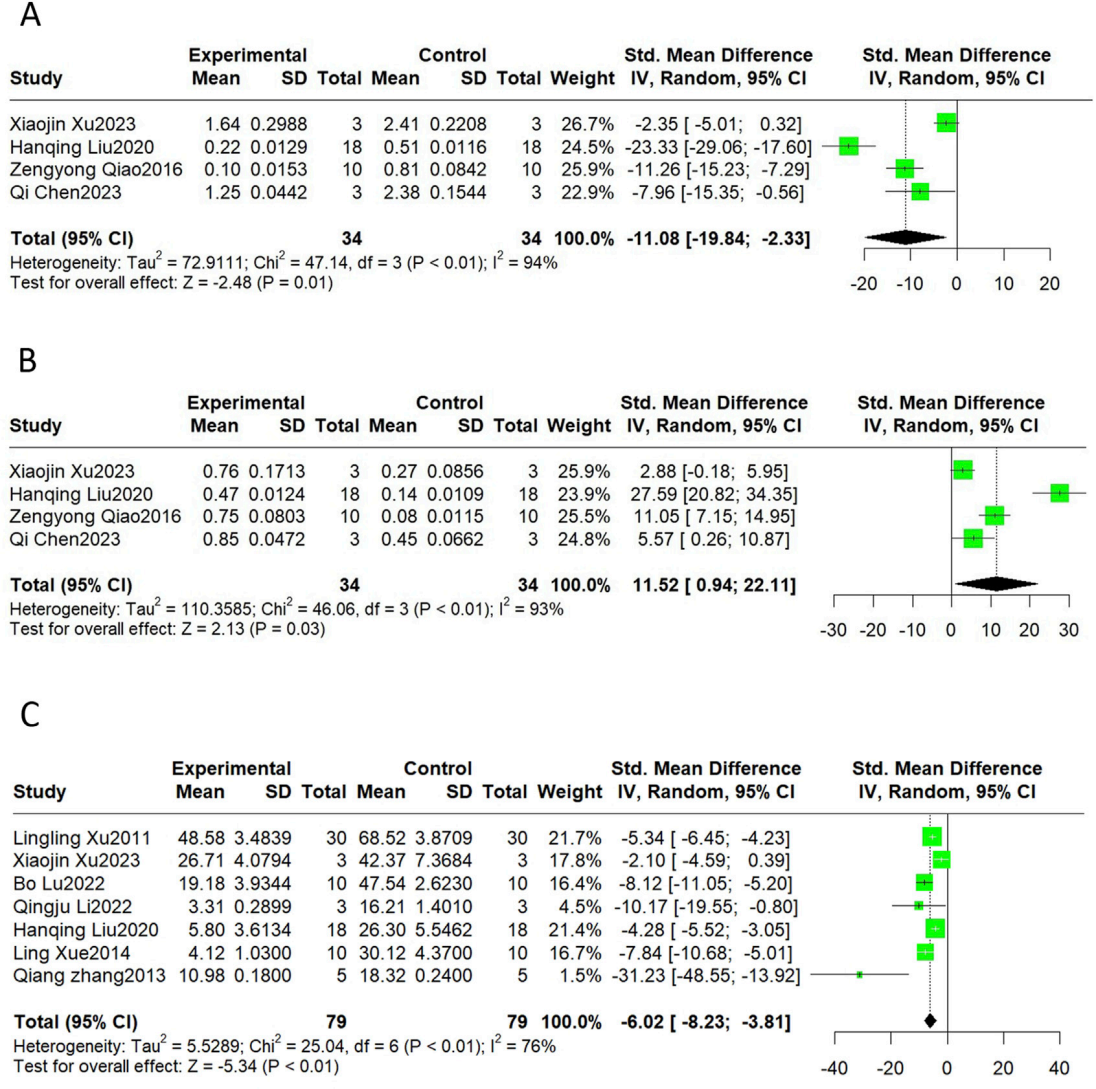
The forest plot of apoptosis indicators: effects of Sal B for decreasing Bax **(A)** and TUNEL **(C)** and increasing Bcl-2 **(B)** compared with the control group.

Bax and Bcl-2 are markers of apoptosis, and the TUNEL assay is commonly used to detect apoptosis ratios. The results revealed that SalB significantly decreased Bax (SMD = −11.08, 95%CI(-19.84, −2.33), *p* < 0.01, I^2^ = 94%) and elevated Bcl-2 (SMD = 11.52, 95%CI(0.94, 22.11), *p* < 0.01, I^2^ = 93%). The level of apoptosis detected by the TUNEL also declined compared to the control group (SMD = −6.02, 95%CI(-8.23, −3.81), *p* < 0.01, I^2^ = 76%) (Figure 8).

### 3.5 Publication bias

Publication bias is a phenomenon in which the results of an experiment determine the likelihood of publication, usually leading to an overestimation of positive results. Funnel plots and Egger’s regression tests were used to analyze the publication bias of infarction size. Visual inspection of the funnel plots revealed a large amount of asymmetry, suggesting potential publication bias, as confirmed by Egger’s test (*p* < 0.05) (Supplementary Figures 3A, B). Trim-and-fill methods were used to correct for potential publication bias (Supplementary Figure 3C). Although trim-and-fill analyses resulted in corrected novel effect sizes (SMD = −2.27, 95%CI(-4.46, −0.09), *p* = 0.04), the association between Sal B and infarct size remained statistically significant.

## 4 Discussion

### 4.1 Summary of evidence

**FIGURE 9:**
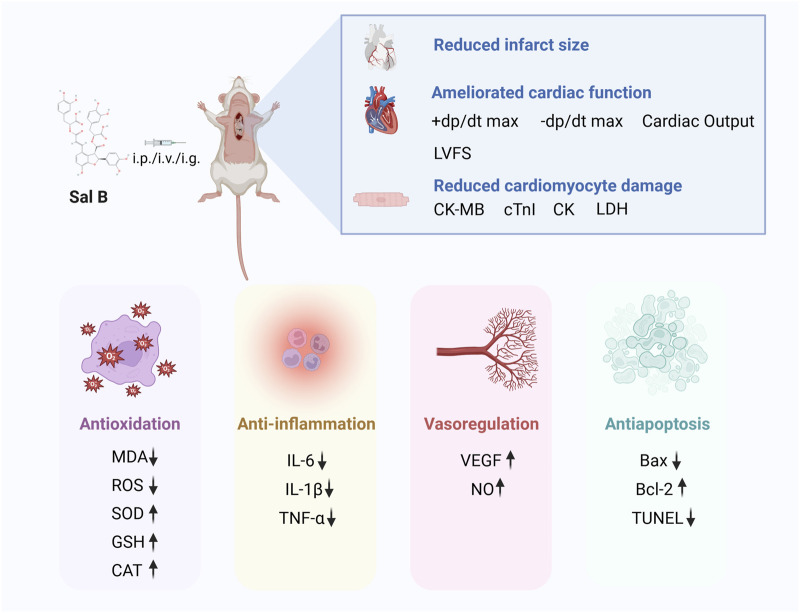
Overview of Sal B protection of MI/R injury.

To the best of our knowledge, this is the first preclinical systematic review and meta-analysis investigating the effects of SalB on MI/R. Thirty-two studies involving a total of 32 papers involving 732 animals were included. The meta-analysis showed that Sal B has a protective effect on the myocardium after ischemia-reperfusion injury and a positive effect on reducing infarct size, improving cardiac function, and reducing myocardial injury. The cardioprotective effects of SalB were mediated by attenuating oxidative stress and inflammation, promoting neovascularization, modulating vascular function, and attenuating cardiomyocyte apoptosis (Figure 9). A high degree of heterogeneity was observed among the included studies; further subgroup and sensitivity analyses were performed. The results of the subgroup analyses showed that the dose of the drug administered could be a potential source of heterogeneity. In contrast, the differences between subgroups for the time of administration, mode of administration, and modality of modeling were not statistically significant. They did not significantly reduce the heterogeneity across subgroups within the group. There was some publication bias among the included studies. In conclusion, Sal B has a clear protective effect against MI/R; however, the exact magnitude of the cardioprotective effect of Sal B should be interpreted with caution.

### 4.2 Possible mechanism

**FIGURE 10:**
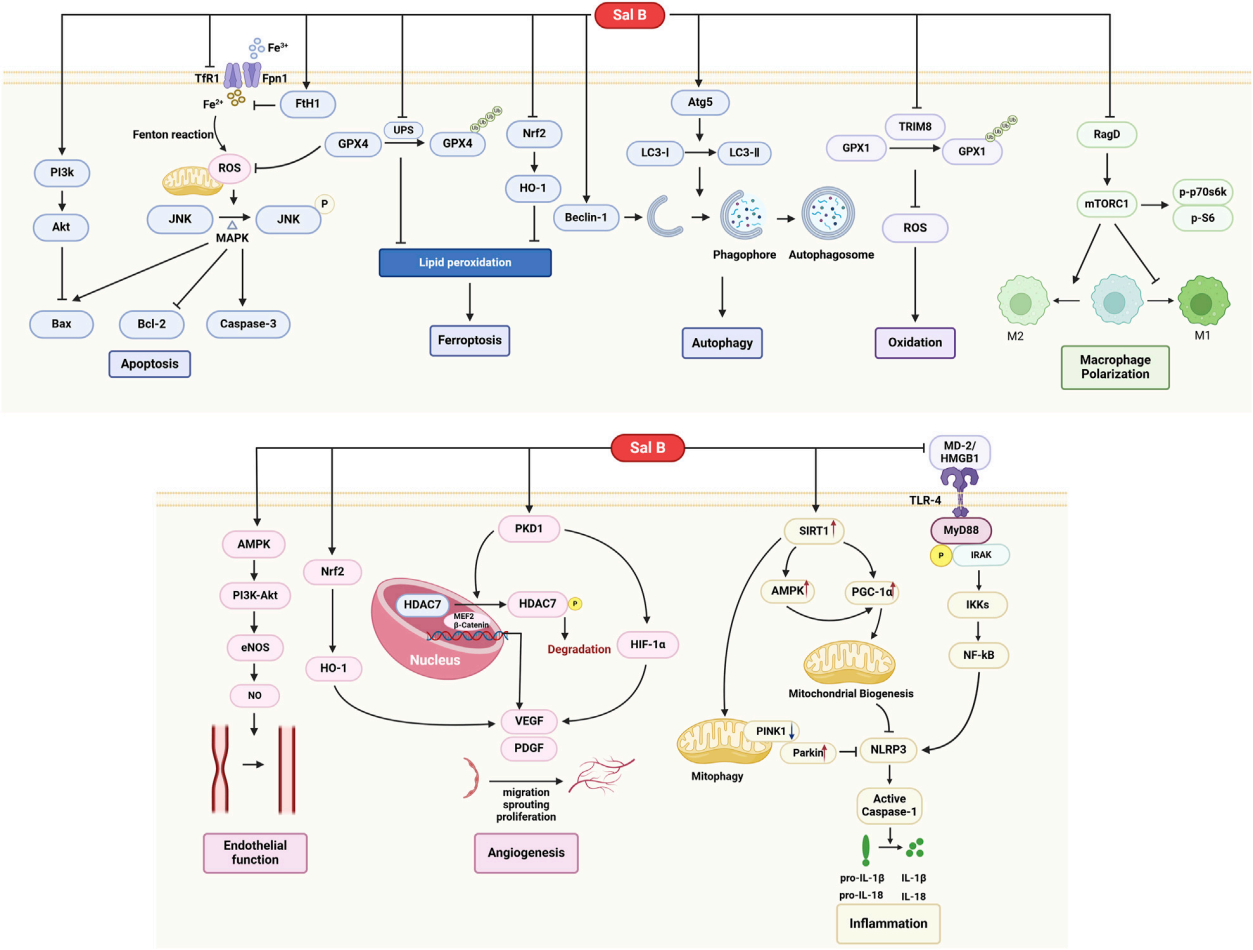
A schematic representation of cardioprotective mechanisms of Sal B for MI/R injury.

MI/R injury involves multiple pathological processes, such as cell death (including apoptosis, autophagy, and ferroptosis), oxidative stress, inflammatory response, vascular endothelial dysfunction, and neovascularization (Ibáñez et al., 2015). There is no satisfactory therapeutic approach in the current clinical practice, and multi-targeted small-molecule drugs may be a solution. Sal B is a promising small-molecule drug for treating MI/R. However, the progression of Sal B from basic research to clinical practice has been hampered by the lack of a comprehensive understanding of its underlying mechanisms. To gain a more comprehensive understanding of the role of Sal B in MI/R, its specific mechanisms and pathways are summarized in this review. In brief, Sal B exerted antioxidant, anti-inflammatory, and modulatory effects on vascular function and regulated cell death (Figure 10).

SalB protects the heart by regulating cardiomyocyte death. Various pathways regulate cardiomyocyte death during MI/R, including apoptosis, ferroptosis, and autophagy, among others (Xiang et al., 2024). Cardiomyocyte apoptosis is a genetically regulated, actively ordered, and self-terminating process involving various enzymes. Caspase-3, Bcl-2, and BAX are important regulators of apoptosis. Caspase-3 is at the crossroads of apoptosis-related signaling pathways. Activating caspase-3 promotes downstream protease cascades that irreversibly amplify apoptotic signaling pathways. Bcl-2 exerts antiapoptotic effects and prevents apoptosis at multiple levels. Bax belongs to a class of pro-apoptotic proteins that promote the subsequent apoptotic cascade by disrupting the outer mitochondrial membrane (Del et al., 2019). Several studies have shown that Sal B can regulate the expression of Caspase-3, Bcl-2, and Bax and thus exert anti-apoptotic effects on cardiomyocytes. Furthermore, several studies have explored the pathways upstream of these three apoptotic regulators. Sal B inhibited mitochondrial ROS accumulation, which consequently inhibited the downstream JNK/MAPK pro-apoptotic signaling pathway (Xu et al., 2023a). In addition, the PI3K-Akt pathway is also one of the upstream pathways in which Sal B alleviates apoptosis (Liu et al., 2020a).

Ferroptosis is a recently discovered type of regulated cell death (RCD) in recent years (Zhao et al., 2023). The core biochemical features of ferroptosis are iron overload and lipid peroxidation. Iron overload refers to the excessive accumulation of intracellular Fe^2+^. TfR1 and Fpn1 present on the cell membrane are channels for transporting Fe^3+^ and Fe^2+^. FTH1(ferritin heavy chain-1) is a component of ferritin that catalyzes the storage of Fe^2+^, thereby reducing free iron levels (Zhang et al., 2024). FTH1 is often used as a biomarker of ferroptosis. The acidic and highly reducing intracellular environment during the early stages of reperfusion promotes the release of iron and iron from ferritin. Iron uptake, utilization, and recirculation balance are disrupted (Xiang et al., 2024). Free iron ions accumulate and catalyze the Fenton reaction, producing increased ROS. Sal B attenuates intracellular free iron accumulation and reduces mitochondrial ROS by decreasing the expression of TfR1 and promoting high expression of FTH1, thereby protecting the myocardium from ferroptosis (Xu et al., 2023a). Lipid peroxidation is another characteristic of ferroptosis. Glutathione peroxidase 4 (GPX4) is the most important intracellular anti-lipid peroxidase and a central regulator of ferroptosis. In the early and middle stages of myocardial infarction, GPX4 expression is markedly reduced, causing the accumulation of lipid peroxides and ultimately leading to ferroptosis (Han et al., 2023). Sal B reduces ubiquitination-proteasome degradation of GPX4 and attenuates MI/R-induced ferroptosis. It was also found that another pathway by which salt B inhibits lipid peroxidation is the inhibition of the Nrf2/HO-1 pathway (Shen et al., 2022).

Autophagy is a lysosome-dependent degradation pathway (Popov et al., 2023). Autophagic lysosomes play a crucial role in autophagy. It degrades damaged cytoplasmic structures and produces amino acids, free fatty acids, and other protein and energy synthesis substances. Autophagy is beneficial during ischemia in MI/R. Autophagy helps cardiomyocytes adapt to environments such as hypoxia and starvation and delays the occurrence of irreversible cell damage (Dong et al., 2019). However, excessive autophagy during reperfusion can induce the progressive consumption of cellular constituents, leading to autophagic cell death. Thus, moderate autophagy activation is cardioprotective and ensures the availability of energy substrates (Bravo-San Pedro et al., 2017). Autophagy is regulated by specific autophagy-related (Atg) genes, among which Atg5 is involved in the formation of phagocytic vesicle expansion on the one hand and promotes the recruitment of LC3II on the other, contributing to the transition from LC3Ⅰ to LC3Ⅱ. LC3II and LC3II/Ⅰ are key indicators of autophagic activity (Sciarretta et al., 2018). In addition, Beclin1 and its complexes are involved in the formation of the isolation membrane as well as the recruitment of Atg proteins to the autophagosomal membrane, and their elevation marks the initiation of autophagy (Shi et al., 2019). Sal B upregulates autophagy lysosomes and the expression of LC3II/Ⅰ and Beclin1 in a myocardial ischemia model in mice induced by ISO. This suggests that Sal B enhances the autophagic activity during myocardial ischemia (Chen et al., 2023). However, whether the effect of Sal B on autophagy during reperfusion is consistent with that during the ischemic process has not yet been investigated.

Oxidative stress is considered one of the major factors that cause myocardial injury in MI/R. During MI/R, particularly during reperfusion, the balance between ROS generation and scavenging is disrupted. A large accumulation of oxygen free radicals damages subcellular membranes and structural systems through several mechanisms (Granger and Kvietys, 2015; Bugger and Pfeil, 2020). Several studies have revealed that Sal B attenuates oxidative stress by reducing ROS production and increasing the expression of the antioxidants SOD, CAT, and GSH(31–34). GPX1 is an important antioxidant enzyme responsible for scavenging ROS accumulated in cells. Ubiquitination and degradation of GPX1 are mediated by TRIM8. Studies have shown that SalB inhibits TRIM8 expression and reduces GPX1 degradation, resulting in accelerated ROS elimination (Lu et al., 2022).

Macrophages regulate the MI/R repair processes. In the early stages of MI/R, M1 macrophages predominate and produce oxidative metabolites and proinflammatory factors. During the following 3–5 days, M2 macrophages gradually replace M1, and M2 macrophages produce anti-inflammatory factors and promote scar formation and angiogenesis (Xu et al., 2023b). Therefore, timely regulation of their transition to the M2 phenotype is essential to ensure cardiac tissue healing and avoid adverse remodeling and systolic dysfunction. Changing the mode of macrophage energy metabolism is one way of changing the polarization direction (Kim et al., 2021). Sal B inhibits the activation of mTORC1 and glycolysis mediated by Rag D, resulting in the inhibition of M1 polarization and an increase in M2 macrophages, ultimately leading to a reduction in myocardial inflammatory factor infiltration and cardioprotective effects (Zhao et al., 2020).

Regulation of vascular endothelial function and promotion of neovascularization are also important for improving MI/R. MI/R injury leads to an imbalance between vasodilatory and vasoconstrictive factors, manifesting as a marked increase in the content of vasoconstrictive factors and a decrease in the release of vasodilatory factors. This imbalance causes vasoconstriction and spasms, leading to worsening of partial ischemia and hypoxia. Sal B can inhibit the release of ET and TXB2 from the vascular endothelium and increase the content of NO and PGI2, thereby regulating endothelial dysfunction and increasing the myocardial blood supply (Wang et al., 2004; Zhang et al., 2008; Yang et al., 2008; Xue et al., 2014). This suggests that this effect may be achieved through the AMPK/PI3k/Akt pathway (Pan et al., 2011). Neovascularization is another approach to repair MI/R damage. VEGF is one of the most widely studied positive regulators of vascular neovascularisation. It also promotes the proliferation and differentiation of vascular endothelial cells. PKD1 is an upstream protein of VEGF that mediates the proliferation, migration, and lumen formation of endothelial progenitors and stem cells. It was found that Sal B induced VEGF synthesis by regulating the PKD1/HDAC axis and the PKD1/HIF-α axis, which promoted neovascularization of ischemic myocardial tissues and increased microcirculatory blood supply in ischemic myocardium (Liu et al., 2020b; Liu et al., 2020c). Additionally, Sal B activates the Nrf2/HO-1 pathway and induces VEGF synthesis, which is involved in the regulation of neovascularization after ischemic injury (Zhou et al., 2018).

The inflammatory response also plays an important role in the myocardial injury caused by MI/R. During ischaemia, inflammation is activated. During reperfusion, restoration of blood flow and oxygen delivery further activates inflammatory signalling pathways. Inflammatory vesicles are sensors that link injury to inflammation. NLRP3 inflammatory vesicles orchestrate the inflammatory response in the absence of pathogens by inducing downstream caspase-1 cleavage and releasing the inflammatory factors IL-1β and IL-18 (Algoet et al., 2023). Sal B promotes the inactivation of NLRP3 inflammatory vesicles via several pathways. First, Sal B can further induce NLRP3 inactivation by inhibiting intracellular ROS production during myocardial ischemia, increasing the mitochondrial membrane potential, regulating the expression levels of SIRT1, Parkin, and PINK1 proteins, and promoting mitochondrial autophagy (Hu et al., 2020). Secondly, Sal B could activate AMPK and PGC-1α-mediated mitochondrial biogenesis via SIRT1, which in turn blocked NLPR3-mediated inflammatory response (Li et al., 2022). Finally, Sal B blocks TLR4 dimerization through competitive chimerism with MD-2 and reduces HMGB1 expression, thereby inhibiting the TLR4/NF-κB pathway, reducing the inflammatory cascade, and attenuating MI/R injury (Hu et al., 2019).

### 4.3 Limitations

This study systematically reviewed the potential efficacy and mechanism of action of SalB in an animal model of MI/R. This systematic review and meta-analysis had several limitations when interpreting the results.

1) The literature search was conducted only on commonly used Chinese and English databases; articles published in other languages may have been ignored. The included studies were conducted in China, hindering our results’ generalizability.
2) Pre-specifying the methodological details of the meta-analysis reduces the risk of inappropriate *post hoc* analyses and selective result reporting (reporting only the results of subgroup analyses showing significant effects) (Bannach-Brown et al., 2024). Therefore, subgroups were established in advance for this meta-analysis. The subgroup analyses for infarct size and LDH levels showed statistically significant differences only between the dose subgroups for myocardial infarct size. Differences between the subgroups regarding administration method, administration time, modeling, and species were not statistically significant. However, no statistical differences between these subgroups should never be interpreted as evidence that the covariates are not related to the effect size (Hooijmans et al., 2022). In addition, the fact that substantial heterogeneity within groups remained after subgroup analyses does not mean that subgroups were not a source of heterogeneity, as these results were obtained under different experimental conditions and need to be investigated in further depth (Hooijmans et al., 2014b).
3) Among the included studies, the duration of administration varied considerably, ranging from one dose to 28 consecutive days. However, considering the diversity of dosing durations in different studies and the limitations of the number of studies, this heterogeneity was not explored, although it may be an important source of heterogeneity.
4) Publication bias was found in this study, which is common in preclinical systematic reviews and meta-analyses (Korevaar et al., 2011; Marks-Anglin and Chen, 2020). Subsequently, this study used the trim-and-fill methods to adjust for publication bias. The results indicated that the direction of the identified effect was reliable. In conclusion, the precise overall effect estimates calculated in this review should be interpreted cautiously, and more high-quality studies are needed to clarify the specific effect sizes.

### 4.4 Implications

In clinical practice, systematic reviews of animal studies can facilitate better scientific research and improve translation, offering the possibility of providing evidence for the potential translational value of animal models for humans (Hooijmans et al., 2018). For future preclinical studies, a systematic review of published studies can reduce unnecessary repetition of costly animal experiments. At the same time, a systematic review and synthesis of evidence can expose biases and deficiencies in the methodology of individual studies, thus reducing misinterpretation of evidence.

The present study systematically reviewed 32 included studies and found that only one study was conducted using female animals, ten did not differentiate between animal sexes, and all other studies included males. However, there were differences in the degree of tolerance to MI/R between the sexes. In animal models of MI/R, females have a smaller infarct size, reduced ischemic systolic dysfunction, and limited fibrotic remodeling compared to males (Medzikovic et al., 2023). Reliable sex subgroup analyses were not possible due to the lack of detailed reporting on the sex of the individual metrics originating from the included studies. Few studies on female animals are currently available, highlighting the need for further studies.

Safe and effective dose ranges are essential for future animal experiments and clinical studies. Interestingly, the different studies’ dosages ranged from 3 to 480 mg/kg; in almost every study, the maximum dose was more effective, with no reports of adverse effects. However, in clinical practice, there is a safe dose range for any drug. A small-sample clinical trial in a Chinese population explored the safe dose of SalB. Adverse events (SAEs) in the single-ascending-dose (SAD) and multiple-ascending-dose (MAD) studies were observed with an incidence of approximately 50% (Cheng et al., 2023). Therefore, further investigation of the optimal SalB dose range of Sal B is required.

The risk of bias assessment showed that the risk of bias for most of the included studies was rated as “unclear” because essential sources of bias, such as allocation sequence concealment, baseline characteristics, and implementation of blinding, were missing from the report. It is difficult to judge the impact of these sources of bias on the results. The lack of methodological details may distort the interpretation of the study results, and there is a risk of overestimating the effect of the intervention. Future animal studies should refine their methodological reporting as much as possible, adhering to the ARRIVE guidelines for reporting animal studies (Percie du Sert et al., 2020).

## 5 Conclusion

Overall, the results of this systematic review and meta-analysis suggest that Sal B has a protective effect against MI/R injury, which may be accomplished through a complex multi-target and multi-pathway mechanism. This study provides an evidence-based assessment and reference evidence for future basic and clinical studies on SalB. Further studies are needed to elucidate the extent to which Sal B exerts its cardioprotective effects and the safety of its use.

## Data Availability

The original contributions presented in the study are included in the article/Supplementary Material, further inquiries can be directed to the corresponding author.
