# Supplementary material for: Protective effect of salvianolic acid B against myocardial ischemia/reperfusion injury: preclinical systematic evaluation and meta-analysis
Source: Front Pharmacol. 2024 Sep 11;15:1452545. doi: 10.3389/fphar.2024.1452545 (PMC11422085; doi:10.3389/fphar.2024.1452545)

Supplementary Material

# Supplementary **Table**: Search strategy

| **Database** | **Search strategy** | **Count** |
| --- | --- | --- |
| Web of science | TS=(“Myocardial Infarction” OR “Myocardial Ischemia” OR “Myocardial Ischemia/Reperfusion Injury” OR “Myocardial I/R” OR “Myocardial I/R Injury” OR “Myocardial Revascularization” OR “Myocardial Reperfusion” OR “Myocardial Stunning” OR “Myocardial Ischemic Preconditioning” OR “Coronary Artery Bypass” OR “Percutaneous Coronary Intervention” OR “Percutaneous Coronary Revascularizations” OR “Percutaneous Transluminal Coronary Intervention”) AND TS=(“Salvianolic acid” OR “Salvianic acid ”) | 174 |
| Pubmed | ("Salvianolic acid" OR "Salvianic acid") AND ("Myocardial Infarction" OR "Myocardial Ischemia" OR "Myocardial Ischemia/Reperfusion Injury" OR "Myocardial I/R" OR "Myocardial I/R Injury" OR "Myocardial Revascularization" OR "Myocardial Reperfusion" OR "Myocardial Stunning" OR "Myocardial Ischemic Preconditioning" OR "Coronary Artery Bypass" OR "Percutaneous Coronary Intervention" OR "Percutaneous Coronary Revascularizations" OR "Percutaneous Transluminal Coronary Intervention") | 105 |
| Embase | #1: "Myocardial Infarction" OR "Myocardial Ischemia" OR "Myocardial Ischemia/Reperfusion Injury" OR "Myocardial I/R" OR "Myocardial I/R Injury" OR "Myocardial Revascularization" OR "Myocardial Reperfusion" OR "Myocardial Stunning" OR "Myocardial Ischemic Preconditioning" OR "Coronary Artery Bypass" OR "Percutaneous Coronary Intervention" OR "Percutaneous Coronary Revascularizations" OR "Percutaneous Transluminal Coronary Intervention":ab,ti  #2: "Salvianolic acid" OR "Salvianic acid":ab,ti  #3: #1 AND #2 | 218 |
| CNKI, Wanfang, VIP | TKA=(丹酚酸 + 丹参多酚酸 + 丹参丹酚酸) AND TKA=(缺血再灌注损伤 + 心肌梗死 + 心肌梗塞 + 心梗 + 再灌注 + 心肌缺血 + 血运重建 + CABG + 冠脉搭桥 + 冠状动脉搭桥 + 冠状动脉旁路移植术 + PCI + 冠脉支架 + 经皮冠状动脉介入术) | CNKI(183), Wanfang(89), VIP(119) |

# Supplementary Figures

**Supplementary Figure 1:** Sensitivity analyses of infarct size.


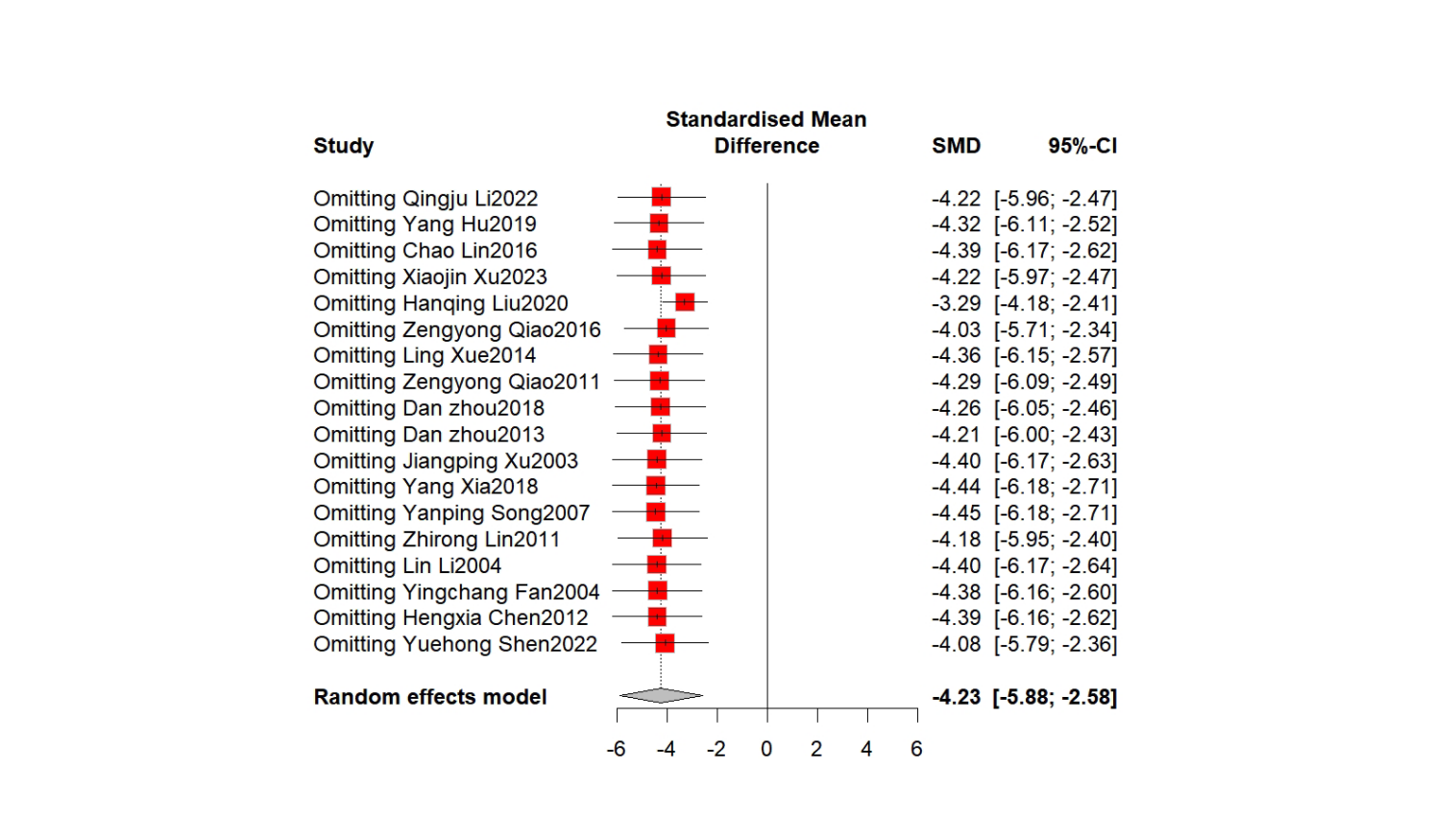


**Supplementary Figure 2:** Sensitivity analysis of LDH.


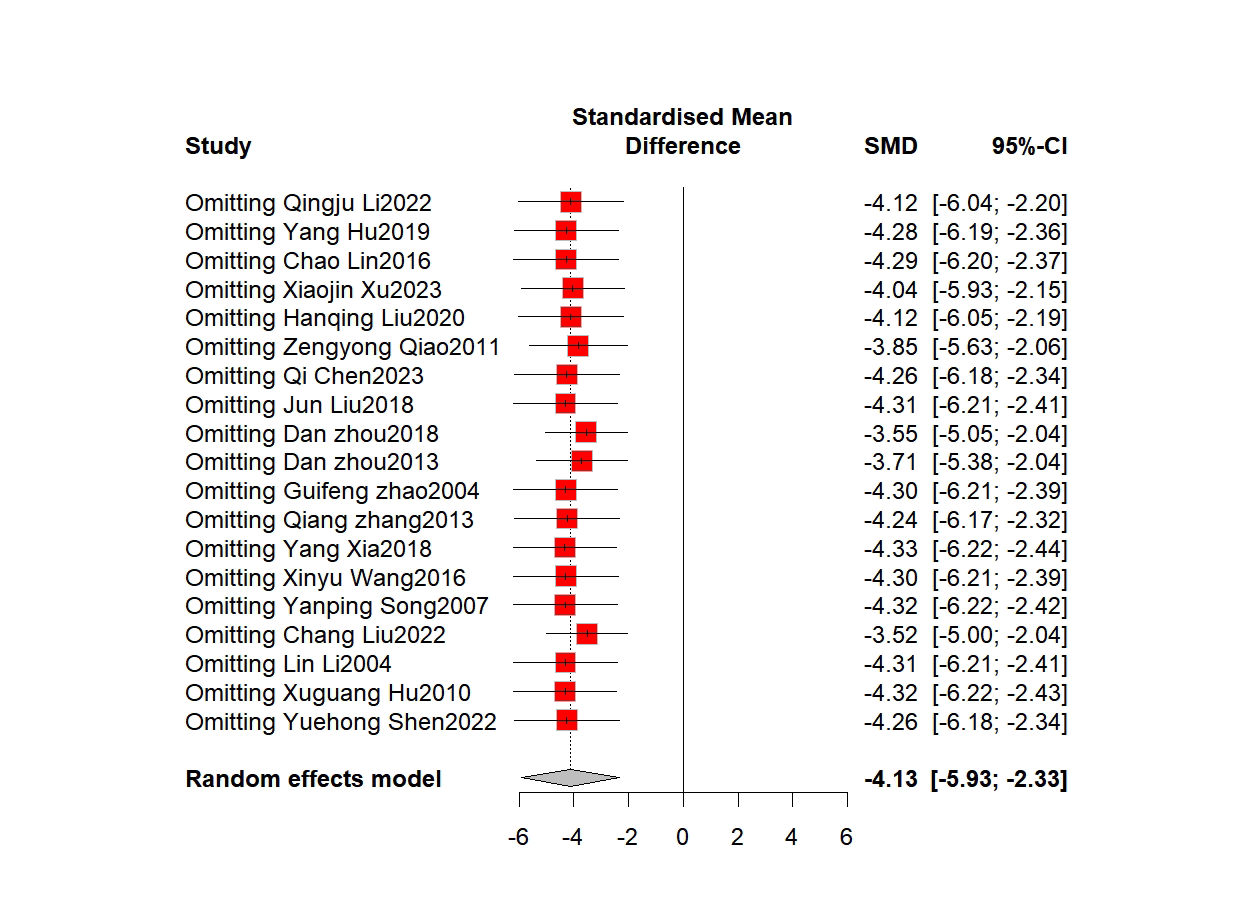


**Supplementary Figure 3:** Funnel plot(A), Egger’s test of Infarct size(B) and Funnel plot of standard error following trim-and-fill method(C).


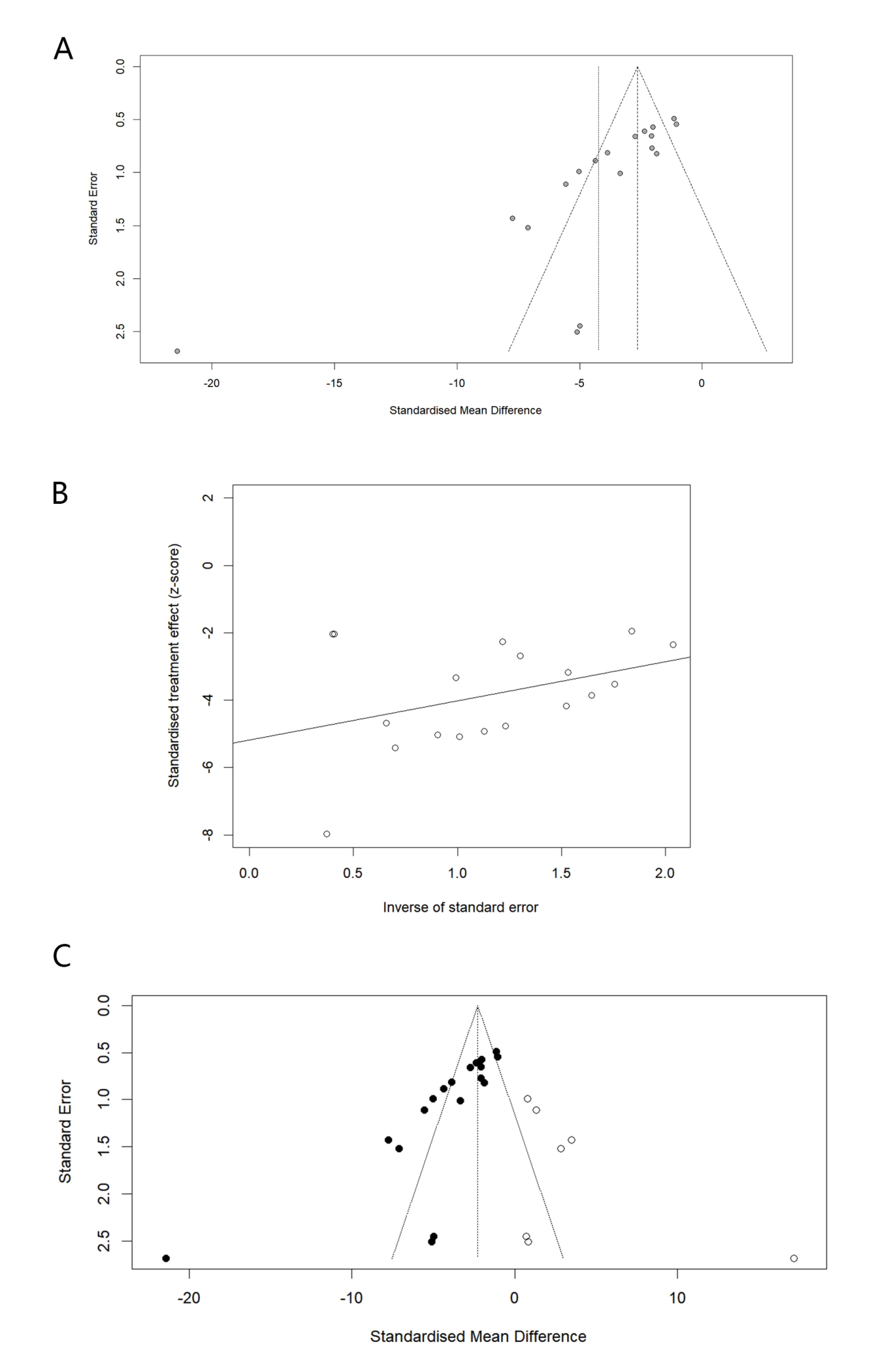

Supplement: Supplementary file 1 [file DataSheet1.docx]
